# Integrative Identification of Deregulated MiRNA/TF-Mediated Gene Regulatory Loops and Networks in Prostate Cancer

**DOI:** 10.1371/journal.pone.0100806

**Published:** 2014-06-26

**Authors:** Ali Sobhi Afshar, Joseph Xu, John Goutsias

**Affiliations:** Whitaker Biomedical Engineering Institute, The Johns Hopkins University, Baltimore, Maryland, United States of America; French National Center for Scientific Research - Institut de biologie moléculaire et cellulaire, France

## Abstract

MicroRNAs (miRNAs) have attracted a great deal of attention in biology and medicine. It has been hypothesized that miRNAs interact with transcription factors (TFs) in a coordinated fashion to play key roles in regulating signaling and transcriptional pathways and in achieving robust gene regulation. Here, we propose a novel integrative computational method to infer certain types of deregulated miRNA-mediated regulatory circuits at the transcriptional, post-transcriptional and signaling levels. To reliably predict miRNA-target interactions from mRNA/miRNA expression data, our method collectively utilizes sequence-based miRNA-target predictions obtained from several algorithms, known information about mRNA and miRNA targets of TFs available in existing databases, certain molecular structures identified to be statistically over-represented in gene regulatory networks, available molecular subtyping information, and state-of-the-art statistical techniques to appropriately constrain the underlying analysis. In this way, the method exploits almost every aspect of extractable information in the expression data. We apply our procedure on mRNA/miRNA expression data from prostate tumor and normal samples and detect numerous known and novel miRNA-mediated deregulated loops and networks in prostate cancer. We also demonstrate instances of the results in a number of distinct biological settings, which are known to play crucial roles in prostate and other types of cancer. Our findings show that the proposed computational method can be used to effectively achieve notable insights into the poorly understood molecular mechanisms of miRNA-mediated interactions and dissect their functional roles in cancer in an effort to pave the way for miRNA-based therapeutics in clinical settings.

## Introduction

MicroRNAs (miRNAs) are small non-coding ribonucleic acids (RNAs) that extensively regulate gene expression in metazoan animals, plants and protozoa. Approximately 22 nucleotides in length, miRNAs usually repress gene expression by binding to sequences with partial complementarity on target messenger RNA (mRNA) transcripts. In mammals, miRNAs are thought to control the activity of more than 60% of all protein-coding genes and extensively participate in the regulation of many cellular functions [1], [2].

With few exceptions, metazoan miRNAs base-pair with their targets imperfectly, following a set of rules that have been formulated by employing experimental and bioinformatics-based analyses [3]. This limited complementarity makes the task of computationally identifying miRNA targets very challenging and usually leads to large numbers of, mostly false, potential targets.

Earlier computational tools have mainly focused on dissecting individual miRNA-target interactions by relying on sequence-based identification of miRNA-target binding sites or on mRNA/miRNA expression data analysis [4]–[6]. Alternative methods use miRNA host genes as proxies for measuring the expression of embedded miRNAs [7] or employ an information-theoretic approach to identify candidate mRNAs that modulate miRNA activity by affecting the relationship between a miRNA and its target(s) [8]. On the other hand, recent work considers co-expression analysis, by assuming that targets of a given miRNA are co-expressed, at least in certain tissues or conditions [9].

Conventionally, many computational methods developed for miRNA-target prediction rely on the assumption that there is an inverse correlation between the expression level of a miRNA and that of its target [10]. However, it has been recently shown that both positive and negative transcriptional co-regulation of a miRNA and its targets are prevalent in the human and mouse genomes [11], [12]. In particular, two types of regulatory circuits (that we will be discussing shortly) have been proposed for miRNA-mediated interactions, which ascribe modulatory and/or reinforcing roles to miRNAs in their networks based on motifs, such as feed-forward loops (FFLs) [13]. As a consequence, miRNA-target predictions solely relying on an inverse correlation assumption are expected to be limited if the prediction method does not appropriately incorporate the underlying FFL network structure.

Based on the previous paradigm, several researchers have investigated the statistical over-representation of network structures involving miRNA and TF co-regulation of mRNAs to identify enriched network motifs and/or assess their prevalence in different biological contexts [14]–[21]. Essentially, these methods compute measures of coordinated gene co-regulation by miRNA and TF regulators. Other investigators have considered regression methods or Bayesian models to quantify statistical associations by determining changes in the expression level of a given mRNA explained by the expression levels of TFs and miRNAs predicted to target the mRNA based on sequence information [22]–[25]. Subsequently, they use the inferred relationships to delineate significant network structures and motifs in a fashion similar to that employed in the aforementioned methods. It is important to note however that the collective findings produced by all these approaches provide further support for the importance of miRNA/TF-mediated FFLs as prevailing network motifs across different biological contexts, reconfirming the hypotheses originally proposed in [11], [12].

In addition to the above, disruptions in gene regulation (for instance, by genetic and epigenetic alterations) believed to induce changes in normal cell function that lead to the progression of pathological conditions, such as cancer, are disseminated through gene regulatory networks. As a consequence, effective treatment of many human diseases may require a fundamental and systemic understanding of genomic regulators, such as miRNAs and TFs, and their networks of interaction. However, systematically inferring molecular interactions by experimental methods is both difficult and costly. Therefore, it is highly desired to develop “reliable” computational approaches capable of identifying such networks. Network predictions can subsequently be used by an expert biologist to formulate novel hypotheses and effectively proceed with their experimental investigation and validation.

Recently, several new methods have been proposed for identifying coordinated miRNA/TF interactions [26], [27]. However, and for a given motif structure (e.g., an FFL), these methods attempt to predict the underlying interactions (the three edges of an FFL) by utilizing limited biological information and a narrow set of computational tools. As a result, although the methods are effective in providing insights into the prevalence of various motif instances in gene regulatory networks, they may not produce reliable predictions from an experimental perspective.

The performance of some of the previous methods has been recently tested in [27]. It was observed that, although some methods were capable of achieving a reasonable success rate in predicting instances of one type of interaction, they were less effective in predicting instances of the other two types, with several algorithms having a success rate of close to or less than 1% in predicting TF-mRNA and TF-miRNA interactions. This highlights the critical fact that predicting pair-wise molecular interactions and constructing higher-order instances of motifs using the predicted edges could translate to higher overall false-positive rates. Since there is a wealth of information on how a TF binds its targets and on their specific regulatory roles, we decided to consider only *experimentally* validated TF-mRNA and TF-miRNA interactions under the FFL framework and shift focus on reliably predicting the poorly understood miRNA-target interaction edge. We believe that, by appropriately constraining the underlying statistical analysis problem, we could potentially increase the reliability of miRNA/TF-mediated gene regulatory loop predictions.

To further constrain the miRNA-target interaction prediction problem, we focus in this paper on certain three-node regulatory motifs. The first set of motifs that our method considers are three-node FFLs that have recently attracted a great deal of attention among systems and experimental biologists. These motifs are excellent models of coordinated miRNA-mediated and transcriptional regulation, which have been hypothesized to be prevalent in the human and mouse genomes [12].

**Figure 1:**
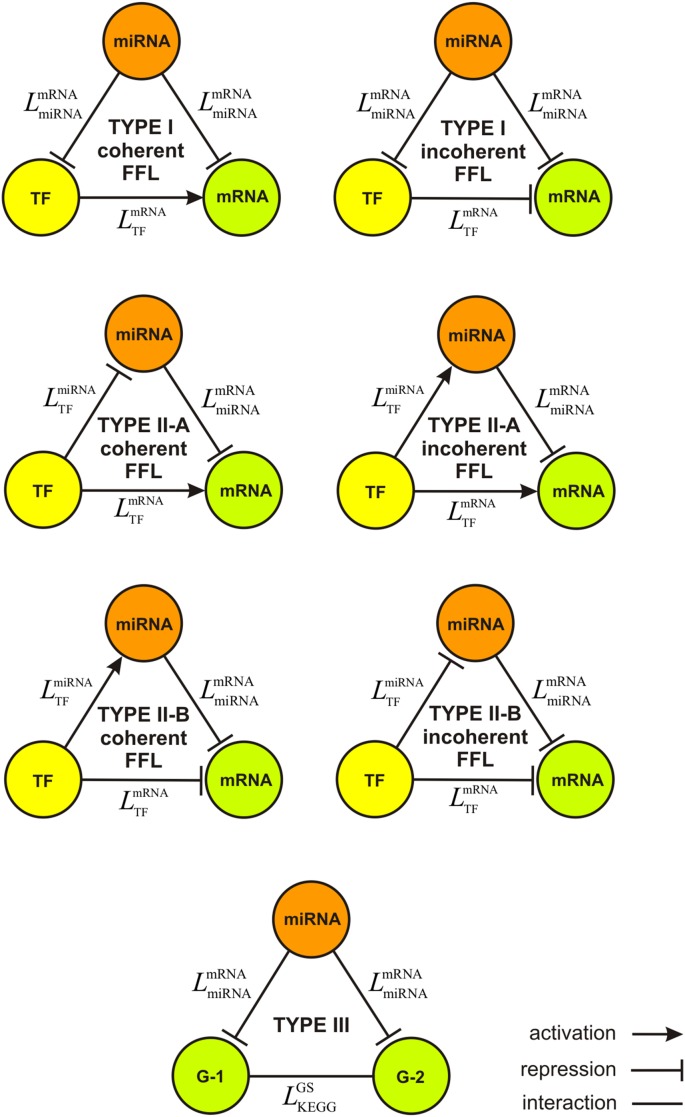
Three-node regulatory motifs considered by IntegraMiR. The Type I FFL consists of triplets (miRNA, TF, mRNA) such that a miRNA simultaneously targets a mRNA and its TF mRNA. The Type II FFL consists of triplets (miRNA, TF, mRNA) such that a TF simultaneously regulates a miRNA and its target mRNA. Finally, the Type III loop consists of triplets (miRNA, G-1, G-2) such that the miRNA simultaneously targets two transcripts in a given KEGG pathway, one from each gene G-1 and G-2, whose corresponding proteins could potentially interact with each other based on a pathway map provided in the KEGG database.

We consider two Type I FFL motifs, in which the miRNA and TF are the upstream and downstream regulators, respectively, as well as four Type II FFL motifs, in which the TF is now the upstream regulator, whereas the miRNA is the downstream regulator – see Figure 1. From a mechanistic perspective, these six FFLs are classified as being *coherent* or *incoherent*. In the coherent case, the miRNA and TF regulators act in a coordinated fashion to reinforce the regulation logic along two feed-forward paths. In Type I and Type II-B coherent FFLs, these paths simultaneously repress the expression of the targeted mRNA. The resulting mechanism is used, for instance, to subdue leaky transcription of a gene by ensuring that its expression stays at an inconsequential level. On the other hand, in a Type II-A coherent FFL, the TF reinforces the transcription of the targeted mRNA by directly activating it as well as by inhibiting its repression by the targeting miRNA regulator.

In the incoherent FFLs, the miRNA and TF regulators act in a coordinated fashion to fine-tune the expression of the targeted mRNA. More specifically, any deviation from the steady-state concentration of the upstream regulator (i.e., the miRNA in Type I and the TF in Type II-A and Type II-B FFLs) would drive the targeted mRNA, as well as the downstream regulator, away from their steady-state levels in the same direction. In this way, the downstream regulator can balance the expression of the targeted mRNA, compensating fluctuations in the expression level of the upstream factor.

Certain cellular processes might be ultra-sensitive to the activity of a given transcript in a specific biological context. In these situations, the “noise buffering” mechanism provided by incoherent FFLs helps maintain target protein homeostasis and ensures that an uncoordinated drift from the steady-state level of the upstream regulator may not result in an undesirable variation in the target protein level which can lead to pathological outcomes. MiRNAs are particularly effective in this setting, owing to their rapid mechanism of action at the post-transcriptional level, as opposed to transcriptional repressors, thus accelerating noise buffering [12].

In addition to the modulatory and/or reinforcing gene regulatory roles that miRNAs are known to play in concert with TFs, they have been hypothesized to play key roles in regulating signaling pathways as well. In this respect, although miRNAs are known to have subtle effects on protein levels of individual targets, their cumulative influence can significantly affect the outcomes controlled by signaling pathways, given the multiplicity of their targets and concurrent downregulation of several of these targets. To take this important aspect into account, our method also considers the basic Type III loop motif depicted in Figure 1, in which a miRNA targets two gene transcripts, G-1 and G-2, whose proteins could potentially interact with each other according to a pathway map provided in the KEGG database (http://www.kegg.jp). The existence of Type III loop motifs is supported by two key hypotheses: (i) miRNAs play major roles in regulating signaling pathways due to their sharp dose-sensitive nature [28]–[32], and (ii) targets of single miRNAs are more connected (i.e., interact) at the protein level than expected by chance [28], [33]–[35].

By comparison, the method proposed in [26] considers only Type II FFLs and does not discriminate between coherent and incoherent FFLs, which is required for a systems-level understanding of transcriptome changes in disease. Moreover, the standard statistical tests used to identify differentially expressed genes between two conditions in a typical gene expression profiling study, as adopted by previous methods [26], [27], become fundamentally flawed in the presence of unaccounted sources of variability (due to biological and experimental factors among others) [36]–[38]. Molecular subtyping information is a critical example of such sources of variability.

To address the previous issues, we develop in this paper IntegraMiR, a novel integrative analysis method that can be used to infer certain types of regulatory loops of deregulated miRNA/TF interactions which appear at the transcriptional, post-transcriptional and signaling levels in a statistically over-represented manner. The proposed method assigns biological roles to miRNAs by integrating five major sources of information together with state-of-the-art statistical techniques to reliably infer specific types of miRNA-target interactions in the context of regulatory loops. In particular, IntegraMiR utilizes:

- mRNA and miRNA expression data.
- Sequence-based miRNA-target information obtained from different algorithms.
- Known information about mRNA and miRNA targets of TFs available in existing databases.
- Certain three-node motifs in gene regulatory networks.
- Known molecular subtyping information available with gene expression data.

To do so, IntegraMiR identifies deregulated miRNAs, TFs and mRNAs by performing statistical analysis within a constrained framework that uses “prior” information comprising recently discovered motifs, available knowledge on miRNA/mRNA transcriptional regulation, and known protein-level interactions on signaling pathways. To illustrate the effectiveness and potential of this method, we apply it on mRNA/miRNA expression data from tumor and normal samples and identify several known and novel deregulated loops in prostate cancer (PCa). This allows us to demonstrate instances of the results and findings in a number of distinct biological settings, which are known to play crucial roles in PCa and other types of cancer.

We should emphasize at this point that IntegraMiR is scalable, in the sense that information from existing or newly developed/updated databases can be input to generate desired/extended results. Moreover, any miRNA/mRNA expression data with samples obtained in any biological context between two conditions can be exploited to infer the corresponding deregulated loops relevant to the particular context at hand. Finally, the interested reader can freely download an R implementation of IntegraMiR from www.cis.jhu.edu/~goutsias/CSS%20lab/software.html.

## Results

### Integrated miRNA/TF-mediated Regulatory Loop Prediction

**Figure 2:**
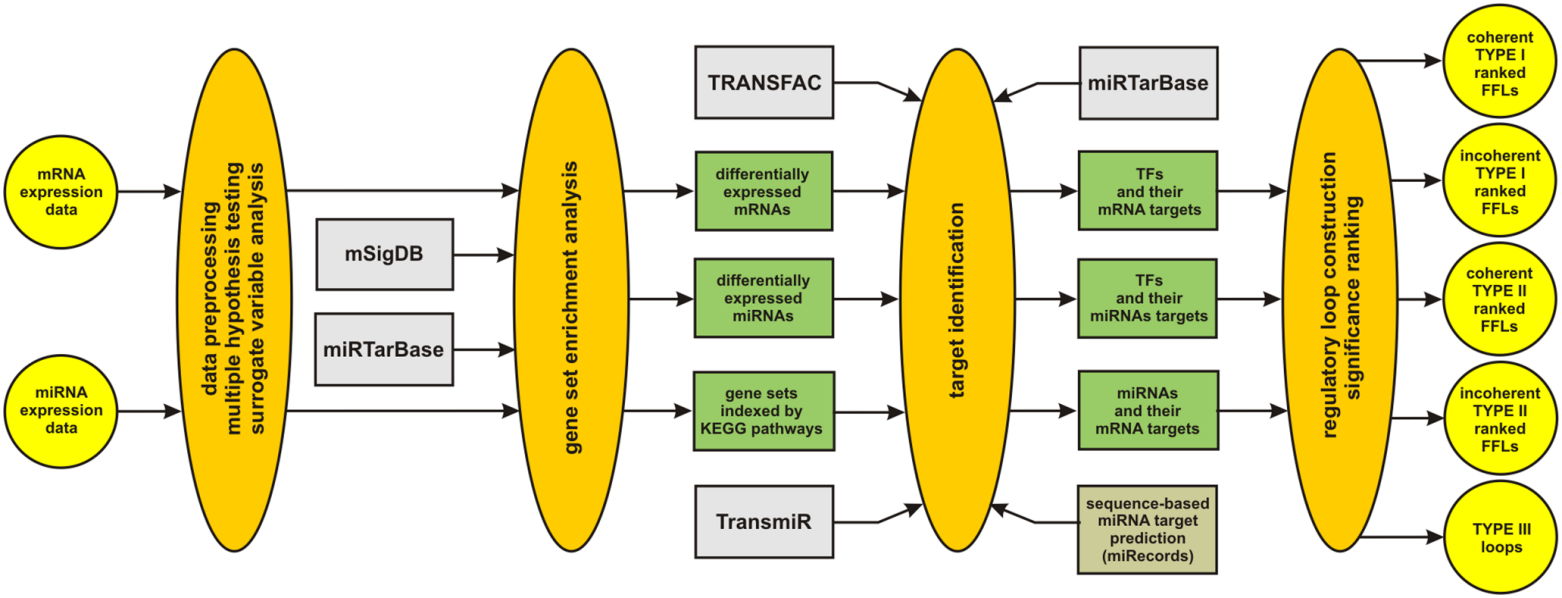
General description of IntegraMiR. The method assigns biological roles to miRNAs by integrating five major sources of information together with state-of-the-art statistical techniques to reliably infer specific types of miRNA-target interactions in the context of regulatory loops from mRNA and miRNA expression data.

The flow-chart depicted in Figure 2 provides a general description of the different steps employed by IntegraMiR. We refer the reader to the “Materials and Methods” section for more details on each step. The procedure uses mRNA and miRNA expression data obtained from prostate tissue at two different biological conditions (normal vs. cancer). It moreover employs results obtained by sequence-based miRNA target prediction algorithms and incorporates information extracted from four databases available online, namely:

–mSigDB (www.broadinstitute.org/gsea/msigdb).
–miRTarBase (http://mirtarbase.mbc.nctu.edu.tw).
–TRANSFAC (www.gene-regulation.com/pub/databases.html).
–TransmiR (http://202.38.126.151/hmdd/mirna/tf).

Note that ENCODE released information recently on TF binding sites based on ChIP-seq experiments for 161 TFs in 91 cell lines (http://genome.ucsc.edu/ENCODE). Unfortunately, this database does not provide the regulation type (activation or repression) of a particular TF-target interaction, information that is critical in our approach. For this reason, IntegraMiR uses TRANSFAC. However, once this information becomes available through ENCODE or any other TF-target database, it can be readily utilized by IntegraMiR.

The first step of IntegraMiR applies standard preprocessing techniques on the raw expression data (such as background correction, normalization, and data heterogeneity correction) to improve data quality, followed by multiple hypothesis testing (MHT) and surrogate variable analysis (SVA) to identify mRNAs and miRNAs that are differentially expressed between the two biological conditions, while correcting for biological variability due to molecular subtyping, multiple testing and batch effects.

The second step implements additional statistical analysis using gene set enrichment analysis (GSEA) to further evaluate the biological significance of certain mRNAs and miRNAs that are not deemed to be differentially expressed by MHT. By employing the molecular signatures database mSigDB of annotated gene sets for use with GSEA and the *experimentally* verified miRNA target database miRTarBase, IntegraMiR constructs three separate groups of gene sets and evaluates the statistical significance of each gene set enriched for deregulation in the available mRNA expression data. The first group consists of gene sets in the mRNA data indexed by a TF mRNA that is not deemed to be differentially expressed by MHT and is determined by mSigDB to directly regulate each gene in the gene set. The second group consists of gene sets in the mRNA data indexed by a miRNA that is not deemed to be differentially expressed by MHT and is determined by miRTarBase to target each gene in the gene set. The third group consists of gene sets in the mRNA data indexed by a specific KEGG signaling pathway [39], [40] included in mSigDB. Finally, TFs associated with statistically significant enriched gene sets are amended to the list of those mRNAs deemed to be differentially expressed by MHT to generate a combined list of differentially expressed mRNAs, and the same is done for miRNAs. We should note here that mSigDB is widely used to obtain gene sets for GSEA analysis. On the other hand, we employ MiRTarBase since this database has accumulated a relatively large number of experimentally validated miRNA-target interactions.

In brief, GSEA determines whether a given set of genes shows statistically significant concordant differences between two biological states [41]. The main reason IntegraMiR applies GSEA after the initial hypothesis testing step is to improve detection of differentially expressed TFs and miRNAs, which may be missed when single expression levels show only moderate changes between the two biological conditions. As a matter of fact, if a number of transcripts are known to participate in a common biological mechanism, then even moderate changes in the expression levels of these transcripts may be statistically significant due to the fact that known biological relationships between transcripts may result in higher statistical power when detecting small variations in their expression levels as compared to the case of single transcripts. Moreover, for certain TFs, TF mRNA expression cannot necessarily be used as a proxy of its activity at the protein level, due to post-transcriptional and post-translational modifications of TFs [42], [43]. To address these issues, IntegraMiR also considers the collective differential expression of genes, as opposed to several procedures followed by other related work discussed earlier that mainly build their analyses on statistics obtained from single transcripts.

The third step of IntegraMiR uses the results obtained by MHT and GSEA, as well as available biological knowledge and sequence-based miRNA target predictions, to identify known *directly* regulated targets of differentially expressed TFs and miRNAs and predicted targets for the miRNAs. By employing the eukaryotic TF database TRANSFAC and the TF/miRNA regulation database TransmiR, IntegraMiR produces a list of differentially expressed TFs together with their gene targets and the regulation type (activation or repression) for each target gene. It also produces a list of differentially expressed TFs together with their differentially expressed miRNA targets and the regulation type for each target miRNA. Note that our choice for using TRANSFAC and TransmiR is based on the fact that TRANSFAC reliably provides the crucial information of regulation type (activation/repression) of a transcription factor and its target gene(s), whereas TransmiR provides the crucial information of the microRNA(s) being regulated by it. On the other hand, to identify mRNA targets of differentially expressed miRNAs, IntegraMiR employs miRecords (http://mirecords.umn.edu/miRecords), an integrated sequence-based miRNA target prediction tool, as well as miRTarBase, a database of experimentally validated miRNA targets. At this step, IntegraMiR produces a list of differentially expressed miRNAs with the corresponding sequence-based target predictions, amended with experimentally validated mRNA targets from miRTarBase to help identify true-positive and false-negative predictions by using available biological knowledge. In this respect, IntegraMiR incorporates a *predictive* module (exploiting miRecords) and a *non-predictive module* (miRTarBase) to accomplish this task.

The fourth step of IntegraMiR implements a technique, described in the “Materials and Methods” section, to construct deregulated loops of the types depicted in Figure 1 using the results obtained from the previous steps. IntegraMiR constructs the following three types of regulatory loops:

(i) An FFL comprising a miRNA which simultaneously targets a TF and a mRNA that is directly regulated by the TF.
(ii) An FFL comprising a TF which directly regulates a miRNA and a mRNA that is directly targeted by the miRNA.
(iii) A regulatory loop comprising a miRNA which simultaneously targets two different genes in a given KEGG pathway whose proteins could potentially interact with each other based on a pathway map provided in the KEGG database.

**Figure 3:**
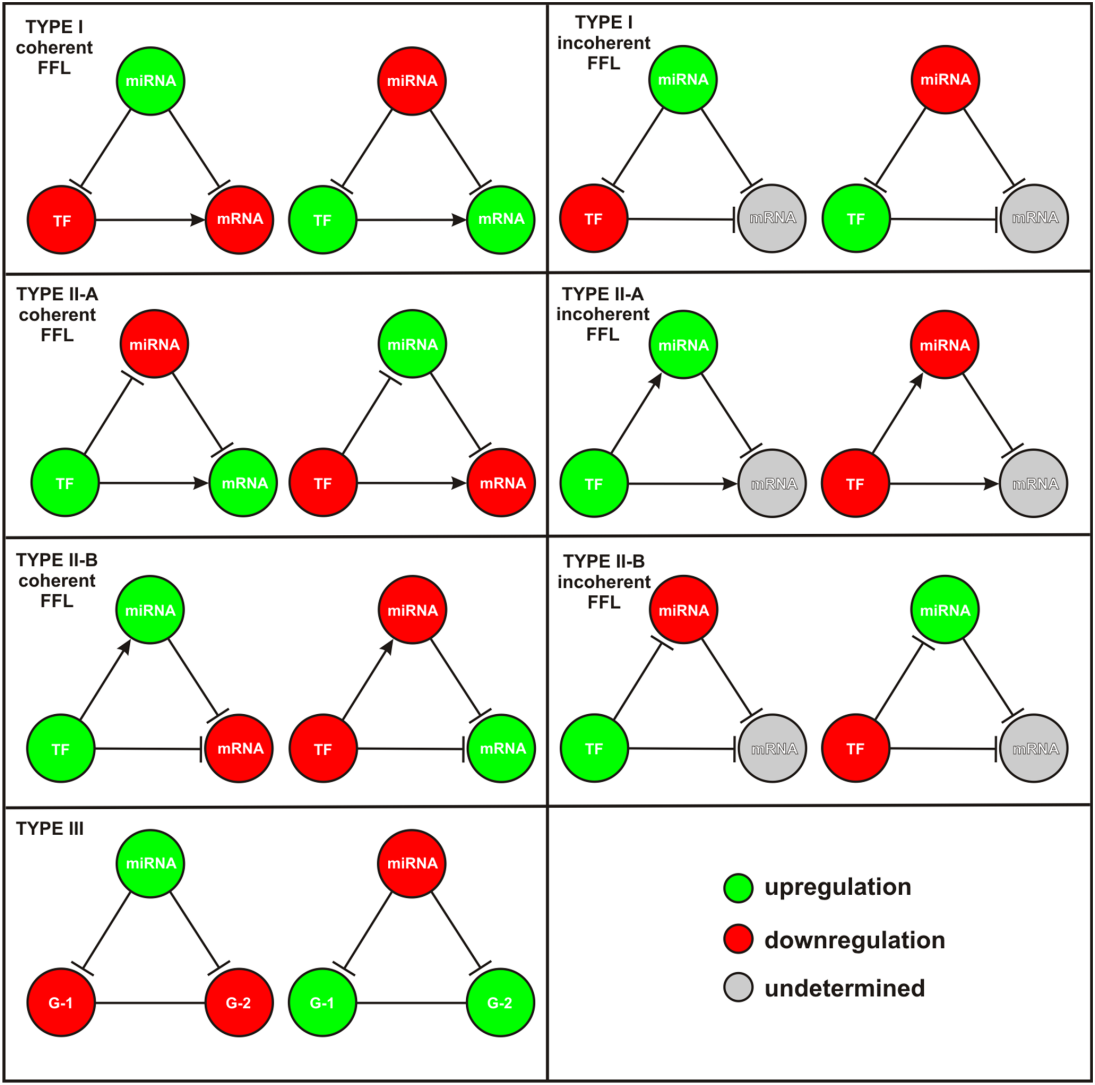
Consistency of deregulated loops. A deregulated loop is deemed to be *consistent* if the expression pattern of its nodes are in agreement with its regulatory edge structure. Any deregulated loop that does not satisfy this property is said to be *inconsistent*.

To rank the constructed regulatory loops in terms of their “significance,” IntegraMiR applies a hypothesis testing procedure using Fisher’s method [44]. The procedure employs Fisher’s summary test statistic, given by Eq. (2) in the “Materials and Methods” section, to combine the MHT-computed *P* values assigned to each node of the loop into one *P* value used as a ranking score for the entire loop. This does not apply to Type III loops, since these loops involve genes and not specific mRNA transcripts. Since the functional roles of regulatory loops are different, IntegraMiR groups these loops into five distinct categories: Type I coherent FFL, Type I incoherent FFL, Type II coherent FFL, Type II incoherent FFL, and Type III loops – see Figures 1 & 2. To provide additional flexibility in interpreting the results, IntegraMiR sorts Type II FFLs into two distinct subgroups, Type II-A and Type II-B, although this additional sorting may not be necessary. Within each group and subgroup, IntegraMiR ranks the deregulated loops by increasing scores, with lower scores corresponding to higher “significance,” and highlights those loops discovered to be deregulated in a manner *consistent* with the underlying edge structure and the expression data, as determined by the rules depicted in Figure 3 (see also the “Materials and Methods” section). It moreover marks miRNA targets depending on whether these targets are predicted by the procedure or have been experimentally validated according to miRTarBase, or both. Note that “consistency” refers to the fact that the expression patterns of the nodes of a deregulated loop are in agreement with its regulatory edge structure. For example, a Type I coherent FFL is said to be consistently deregulated if it comprises an upregulated miRNA and downregulated TF and mRNA, or a downregulated miRNA and upregulated TF and mRNA; see Figure 3.

### IntegraMiR Identifies Extensive Transcriptional, Post-transcriptional and Signaling Deregulation in PCa

To investigate the effectiveness of IntegraMiR in delineating miRNA-mediated regulatory loops, we use mRNA microarray expression data, obtained from 48 normal and 47 prostate tumor tissue samples (NCBI GEO database, accession number GSE29079), as well as miRNA microarray expression data obtained from matched normal and cancerous tissue samples, extracted from 20 individuals (NCBI GEO database, accession number GSE23022). For more information about this data, we refer the reader to the “Materials and Methods” section. After data preprocessing, IntegraMiR incorporates Surrogate Variable Analysis (SVA) [36], together with MHT, to identify differentially expressed genes between the two conditions. It has been shown that SVA increases the biological accuracy and reproducibility of analyses in genome-wide expression studies [36], [37]. IntegraMiR employs SVA to take into account biological variabilities due to molecular subtypes categorized by the status of TMPRSS2-ERG gene fusion, which has been identified in about half of all PCa cases and is a critical early event in the development and progression of this disease [45]–[47].

IntegraMiR first performs MHT, using a moderated t-statistic [48], to separately identify mRNAs and miRNAs that are differentially expressed between tumor and normal samples. This analysis identifies extensive transcriptional deregulation in the tumor tissue samples: 7,934 genes (out of 17,324) are found to be differentially expressed based on their statistical significance, with 164 of these genes being overexpressed by a fold change 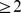 or repressed by a fold change 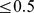
– see Tables S1 & S2. The gene list we provide in Table S2 contains important genes, such as TARP, MYC, SNAI2 (SLUG), WIF1 and ERG among others, which have been previously characterized in PCa.

Analysis of the corresponding miRNA expression data by MHT results in 18 (out of 847) differentially expressed human miRNAs, which we list in Table 1 (first 18 miRNAs) – see also the Table S3. Recently, deep sequencing analysis of miRNA expression profiles identified 33 miRNAs as being differentially expressed in PCa, with miR-375, miR-200c, miR-143 and miR-145 exhibiting the most pronounced deregulation [49]. We compared the IntegraMiR results to the ones obtained by deep sequencing. Of the 18 miRNAs identified by IntegraMiR, 7 miRNAs (miR-200c, miR-20a, miR-375, miR-106a, let-7a, miR-21, and miR-106b) have been confirmed to be upregulated by deep sequencing analysis, whereas 2 miRNAs (miR-221 and miR-145) have been confirmed to be downregulated. The remaining 9 miRNAs identified by MHT were not detected by deep sequencing.

**Table 1:** Differentially expressed miRNAs identified by IntegraMiR.

Rank	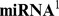	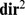	FDR	FDR
			(MHT)	(GSEA)
1	miR-222	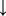	6.58E-4	n/a
2	**miR-200c**	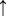	1.32E-3	n/a
3	**miR-221**	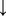	1.34E-3	n/a
4	**miR-20a**	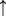	1.70E-3	n/a
5	miR-20b	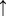	2.55E-3	n/a
6	miR-182	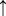	3.52E-3	n/a
7	**miR-375**	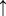	3.63E-3	n/a
8	miR-17	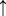	4.12E-3	n/a
9	miR-93	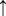	7.64E-3	n/a
10	**miR-145**	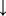	9.58E-3	n/a
11	**miR-106a**	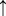	1.04E-2	n/a
12	miR-141	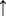	2.05E-2	n/a
13	mir-720	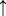	2.27E-2	n/a
14	**let-7a**	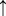	2.83E-2	n/a
15	miR-214	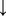	2.85E-2	n/a
16	miR-200b	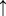	2.95E-2	n/a
17	**miR-21**	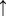	2.95E-2	n/a
18	**miR-106b**	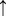	4.66E-2	n/a
19	**miR-125b**	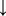	3.15E-1	9.02E-4
20	**miR-143**	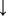	7.45E-1	1.06E-1
21	**miR-29a**	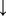	8.62E-1	1.06E-1
22	**miR-24**	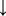	8.79E-1	1.06E-1
23	**miR-199a**	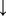	9.96E-1	1.06E-1

1 Highlighted miRNAs have been confirmed by deep sequencing analysis.

2 Direction of deregulation.

During the second step of IntegraMiR, application of GSEA on gene sets of TF targets obtained from mSigDB discovers 37 significantly deregulated TFs, which are not detected by the initial MHT step based on single gene analysis. We list these TFs in Table S4. Interestingly, several of these TFs (e.g., NKX3-1, SMAD1/3, SRF, ETV4 and ELK1) are known to play important roles in PCa, as well as in other types of cancer.

Likewise, application of GSEA on gene sets of experimentally validated (by deep sequencing analysis) miRNA targets obtained from miRTarBase identifies 5 significantly downregulated miRNAs, which are not detected by MHT. We list these miRNAs in Table 1 (last 5 miRNAs). In both cases, and for each TF or miRNA, GSEA is performed based on the availability of gene sets in the data.

Finally, application of GSEA identifies 30 significantly deregulated signaling pathways, among the 186 KEGG signaling pathways available in mSigDB. We list the results in Table 2. Among other pathways, the list contains the TGF-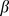 and Wnt Signaling pathways, which have been implicated in PCa initiation and progression. Naturally, the results also include the Prostate Cancer and Adherens Junction pathways. The last pathway regulates intercellular adhesion that plays an important role in epithelial-to-mesenchymal transition (EMT), considered to be an important step in tumor progression [50], [51]. In the following, we limit our results and discussions to miRNA-target interactions associated with these four pathways.

**Table 2:** Significantly deregulated KEGG signaling pathways identified by IntegraMiR.

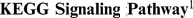	FDR (GSEA)
DILATED_CARDIOMYOPATHY	6.67E-4
ARRHYTHMOGENIC_RIGHT_VENTRICULAR_CARDIOMYOPATHY_ARVC	6.67E-4
REGULATION_OF_ACTIN_CYTOSKELETON	8.34E-4
HYPERTROPHIC_CARDIOMYOPATHY_HCM	8.34E-4
**TGF_BETA_SIGNALING_PATHWAY**	3.68E-3
CALCIUM_SIGNALING_PATHWAY	4.09E-3
FOCAL_ADHESION	8.16E-3
ECM_RECEPTOR_INTERACTION	8.16E-3
**WNT_SIGNALING_PATHWAY**	8.77E-3
MAPK_SIGNALING_PATHWAY	1.40E-2
PROPANOATE_METABOLISM	1.55E-2
VALINE_LEUCINE_AND_ISOLEUCINE_DEGRADATION	1.76E-2
PHOSPHATIDYLINOSITOL_SIGNALING_SYSTEM	1.76E-2
FC_GAMMA_R_MEDIATED_PHAGOCYTOSIS	4.02E-2
PATHWAYS_IN_CANCER	4.36E-2
VASCULAR_SMOOTH_MUSCLE_CONTRACTION	4.36E-2
AXON_GUIDANCE	8.86E-2
UBIQUITIN_MEDIATED_PROTEOLYSIS	1.00E-1
MELANOGENESIS	1.00E-1
**PROSTATE_CANCER**	1.00E-1
ONE_CARBON_POOL_BY_FOLATE	1.20E-1
INOSITOL_PHOSPHATE_METABOLISM	1.49E-1
VASOPRESSIN_REGULATED_WATER_REABSORPTION	1.68E-1
**ADHERENS_JUNCTION**	1.71E-1
LONG_TERM_POTENTIATION	1.71E-1
PURINE_METABOLISM	1.71E-1
GLYCINE_SERINE_AND_THREONINE_METABOLISM	1.72E-1
GAP_JUNCTION	1.92E-1
ARGININE_AND_PROLINE_METABOLISM	2.32E-1
MELANOMA	2.50E-1

1 Highlighted pathways used by IntegraMiR to construct Type III loops.

**Figure 4:**
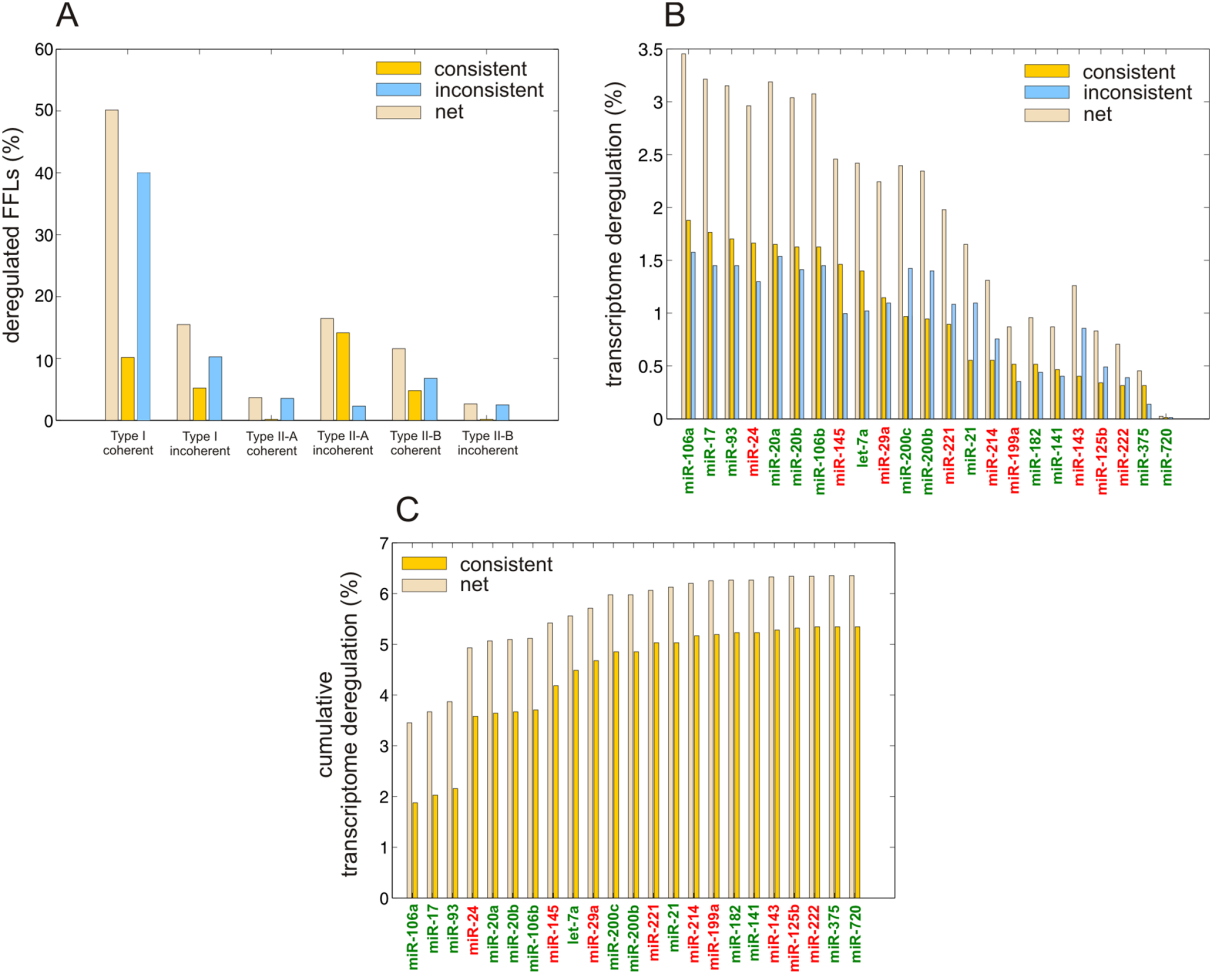
Predicted FFL-based transcriptome deregulation in PCa. (A) Distribution of the fraction of deregulated FFL subtypes grouped in terms of consistent and inconsistent deregulation based on expression data. (B) Percentages of transcriptome change due to significantly upregulated (in green) and downregulated (in red) miRNAs. (C) Cumulative percentages of transcriptome change due to significantly upregulated (in green) and downregulated (in red) miRNAs.

Lastly, and during the third and fourth steps, IntegraMiR constructs deregulated regulatory loops, sorts them into the seven groups depicted in Figure 1 and ranks the Type I and Type II FFLs within each group using the scores computed by Fisher’s summary test statistic. IntegraMiR predicts a large number of deregulated Type I and Type II FFLs, which we list in Tables S5–S10 (see also Figure 4A): 2,104 Type I coherent, 649 Type I incoherent, 154 Type II-A coherent, 690 Type II-A incoherent, 486 Type II-B coherent, and 111 Type II-B incoherent. Moreover, the method predicts a large number of deregulated miRNA-target interactions that could potentially form Type III loops, which we list in Table S11: 904 miRNA-mRNA pairs in the TGF-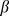 Signaling Pathway, 1,611 miRNA-mRNA pairs in the Wnt Signaling Pathway, 1,025 miRNA-mRNA pairs in the Prostate Cancer Pathway, and 896 miRNA-mRNA pairs in the Adherens Junction Pathway.

### IntegraMiR Reveals Appreciable FFL-based Transcriptome Deregulation

To gain insight into the occurrence of deregulated Type I and Type II FFLs, we depict in Figure 4A the fractions of deregulated FFL subtypes (among all deregulated FFLs predicted by IntegraMiR) grouped in terms of consistent and inconsistent deregulation (as defined in the “Materials and Methods” section and illustrated in Figure 3) based on expression data. The results suggest that certain FFL subtypes contribute to a larger portion of the observed net FFL deregulation than other subtypes. Interestingly, consistent FFL deregulation accounts for about 35% of net FFL deregulation. This type of deregulation is important since its functional characteristics are corroborated by the available expression data, which provides a first level of evidence of their significance. For this reason, an experimentalist may want to first consider this type of FFL deregulation for validation. Among the consistently deregulated FFLs, the Type II-A incoherent FFLs account for about 14% of net FFL deregulation, followed by Type I coherent FFLs, which account for 10%. On the other hand, Type I-A incoherent and Type II-B coherent FFLs each account for about 5% of net FFL deregulation, whereas, the two remaining subtypes, Type II-A coherent and Type II-B incoherent, account for less than 1%. It is striking however that 40% of FFL deregulation is attributed to inconsistent deregulation of Type I coherent FFLs. Inconsistent FFL deregulation suggests that the implied molecular interactions between the three nodes (miRNA, TF, mRNA) of a particular FFL may not be used to explain biological function on its own, based on the transcript levels of the nodes in the expression data. In this case, further investigation of underlying biological mechanisms that could affect the three FFL nodes is needed, including other FFLs sharing a node with the particular FFL under consideration.

To explain the previous result, note that we expect in the coherent case to observe a relatively smaller number of consistently than inconsistently deregulated FFLs since, for a coherent FFL to be consistently deregulated, the abundance of the three associated molecular species (miRNA, TF, and mRNA) must satisfy the rules depicted in Figure 3 (see also the “Materials and Methods” section). The required conditions however may not be observed in the data, since the abundance of a molecular species may be influenced by several FFLs or by factors other than FFL regulation. Clearly, the results depicted in Figure 4A corroborate this remark. On the other hand, IntegraMiR predicts that Type I coherent FFL deregulation accounts for an appreciable portion (50%) of net FFL deregulation which, together with the previous remark, explains the high percentage (40%) of net FFL deregulation due to inconsistently deregulated Type I coherent FFLs.

By examining the constituent interactions that form deregulated FFLs, we determined, for each significantly deregulated miRNA, the percentage of transcriptome deregulation attributed to that miRNA. The results are depicted in Figure 4B, ranked in terms of decreasing percentages of consistent deregulation. We call a miRNA-target interaction to be *consistent*, if the miRNA and the associated mRNA target exhibit anti-correlated deregulation in the data. Note that miR-106a is responsible for the most consistent (1.88%) and the most inconsistent (3.45%) transcriptome deregulation, whereas miR-720 has negligible transcriptome changes associated with it. Finally, the cumulative distributions depicted in Figure 4C reveal that 6.35% of transcriptome changes between normal and cancer samples are due to FFLs with significantly deregulated miRNA nodes, with 5.34% of the changes being accounted for by consistently deregulated miRNA-target interactions.

### miRNA-Target Predictions are Consonant with MiRNA Family Co-targeting Hypothesis

Among the top miRNAs depicted in Figure 4B are members of three miRNA clusters that have been investigated in other types of cancers as well [52]: miR-17/92 on human chromosome 13 (with genomic locus 13q31.3) and its two cluster paralogs, miR-106a/363 on chromosome X (Xq26.2) and miR-106b/25 on chromosome 7 (7q22.1). Members of these clusters have been established to play essential roles in the normal development of heart, lungs, and the immune system and are involved in tumor formation with oncogenic roles [53]–[55]. More importantly, miR-17 and miR-20a (from the miR-17/92 cluster), miR-106a and miR-20b (from the miR-106a/363 cluster), as well as miR-106b and miR-93 (from the miR-106b/25 cluster) belong to the same family of miRNAs (i.e., miRNAs with identical seed regions) and are deemed to be significantly upregulated by IntegraMiR. Note however that individual miRNAs on the same cluster could exhibit varied levels of expression and, for some miRNAs, no expression at all in certain cell lines [56], [57]. Along these lines, several miRNAs in the miR-17/92 cluster and its two paralogs (in particular, miR-18a, miR-19a, miR-19b-1 and miR-92a-1 from the miR-17/92 cluster, miR-18b, miR-19b-2, miR-92a-2 and miR-363 from the miR-106b/25 cluster, as well as miR-25 from the miR-106a/363 cluster) are not identified as being differentially expressed based on the expression data we used in this study.

**Figure 5:**
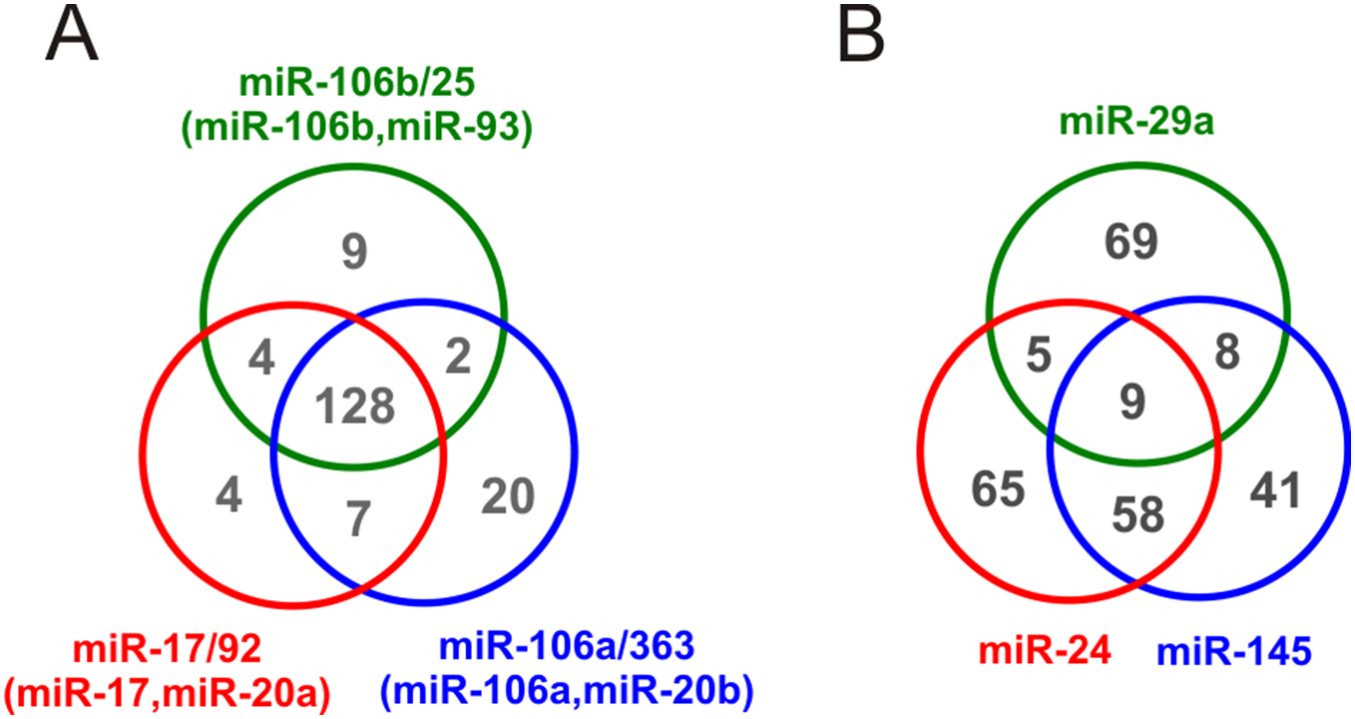
Comparison of miRNA-Target predictions for miRNAs in the same family versus those not in one family. (A) Venn diagram depicting the number of mRNA targets of six significantly upregulated miRNAs, miR-17 and miR-20a (from the miR-17/92 cluster), miR-106b and miR-93 (from the miR-106b/25 cluster), and miR-106a and miR-20b (from the miR-106a/363 cluster), which belong to the same family. (B) Venn diagram depicting the number of mRNA targets of three significantly downregulated tumor suppressor miRNAs, miR-24, miR-29a, and miR-145, which do not belong to one family.

Recent work suggests that members of the same family of miRNAs tend to target common transcripts due to their shared seed sequences [35]. The results obtained by IntegraMiR corroborate this hypothesis. In Figure 5A, we use a Venn diagram to depict the numbers of mRNA targets predicted by IntegraMiR for the previous six miRNAs (obtained from miRNA-target interactions among all FFLs in our results – see Tables S5–S10). Clearly, a high level of overlap exists among the three target sets. In particular, our results predict that all six miRNAs target a set of 128 different mRNAs. This finding has also been observed by using an alternative method and different data sets [9], suggesting that cooperation among the six deregulated miRNAs may be present in other cancer types as well.

On the other hand, the top three miRNAs miR-24, miR-29a, and miR-145 in Figure 4B which were found by IntegraMiR to be significantly downregulated, do not belong to one family and are not known to reside on a common cluster according to the miRBase (www.mirbase.org) database. The results depicted in Figure 5B show that, in this case, the amount of overlap is less pronounced than the one depicted in Figure 5A. It is important to note that these three miRNAs have been hypothesized to possess tumor suppressor roles: miR-24 has recently been shown to suppress expression of two crucial cell cycle control genes, E2F2 and Myc [58], low levels of miR-29a have been attributed to the methylation of its promoter region in PCa [59], and miR-145 is hypothesized to play roles in several types of cancer [60].

### IntegraMiR Predicts Appreciable FFL-based miRNA-TF Co-regulation

**Figure 6:**
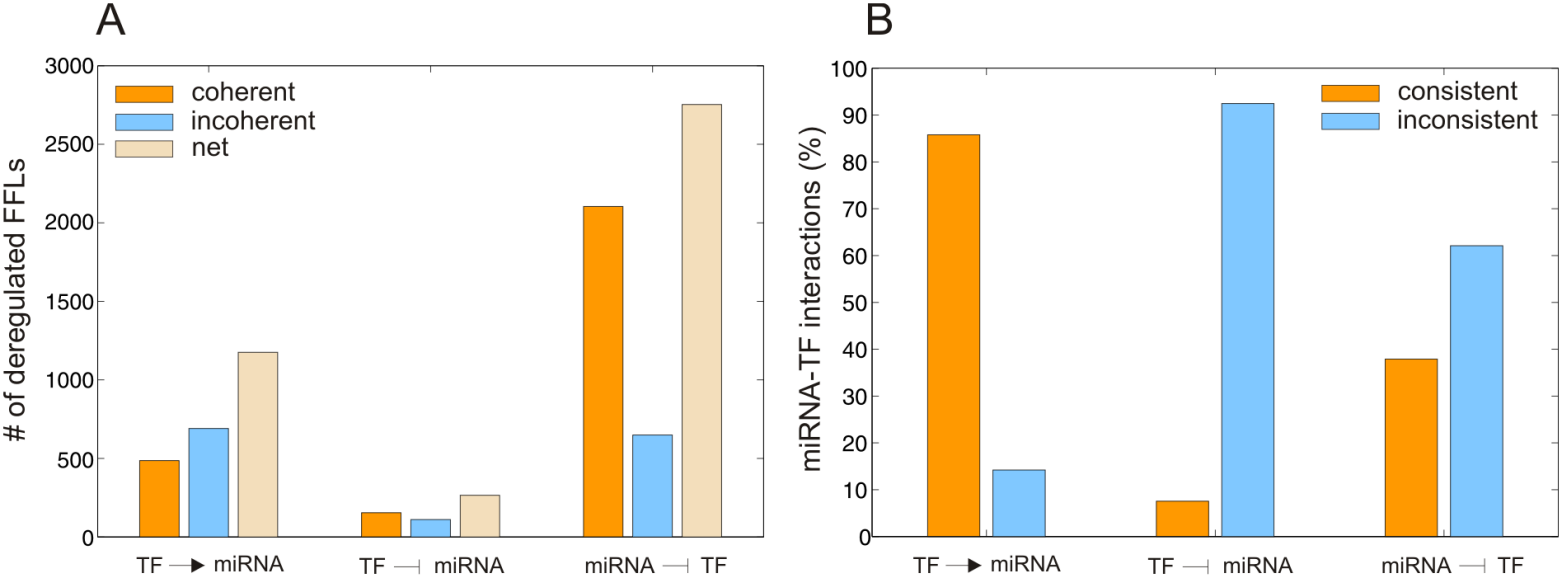
Predicted FFL-based miRNA-TF co-regulation. (A) Numbers of coherent and incoherent deregulated FFLs for each type of miRNA-TF interaction. (B) Percentages of consistently and inconsistently deregulated FFLs under each miRNA-TF interaction type depicted in (A).

We now focus our attention on FFL-based miRNA-TF co-regulation. In Figure 6A, we depict the numbers of coherent and incoherent deregulated FFLs predicted by IntegraMiR for each type of miRNA-TF interaction whereas, in Figure 6B, we depict the percentages of consistently and inconsistently deregulated miRNA-TF interactions under each category. The results suggest that, in PCa, both coherent and incoherent FFLs are deregulated, although the total coherent FFLs outnumber the incoherent ones, an observation that is especially true when the miRNA represses the TF (Type I). Moreover, the most prevalent FFL deregulation involves repression of the TF by the miRNA (Type I coherent and incoherent), followed by FFL deregulation that involves activation of the miRNA by the TF (Type II-A incoherent and Type II-B coherent). On the other hand, deregulation of FFLs that involve repression of the miRNA by the TF (Type II-A coherent and Type II-B incoherent) is not substantial. Note also that consistent deregulation of FFLs that involve activation of the miRNA by the TF (Type II-A incoherent and Type II-B coherent) is appreciably more prevalent than inconsistent deregulation whereas the opposite is true for the case of FFLs in which the TF represses the miRNA.

In Table S12, we list all miRNA-TF pairs associated with the deregulated FFLs predicted by IntegraMiR (obtained from miRNA-TF interactions among all the FFLs in our results – see Tables S5–S10), categorized by their interaction type. As a notable example, the six miRNAs considered in Figure 5A appear in the list as being consistently deregulated together with the MYC oncogene, which acts as their transcriptional activator. We investigated how many of the 128 common mRNAs targeted by these six miRNAs were predicted to form FFLs with MYC. IntegraMiR predicts 79 of the 128 mRNAs to be under the regulatory control of MYC, divided into two sets, with 33 mRNAs being in the first set and 46 mRNAs in the second – see Figure S1. All six miRNAs interact with the first set of mRNAs in Type II-B coherent FFL configuration and with the second set in Type II-B incoherent FFL configuration. Among these mRNAs, APP from the first set and E2F1 from the second set have experimentally validated interactions with these miRNAs according to miRTarBase.

### IntegraMiR predictions lead to bona fide miRNA-mediated regulatory networks

To demonstrate the significance of the results obtained by IntegraMiR from a mechanistic point of view, we focus on two biological settings known to play crucial roles in PCa and other types of cancer. This will help us explain the functional roles of regulatory modules and illustrate how one can use these modules to build an integrated network model for a specific biological setting or molecular species of interest.

#### TP53 miRNA-mediated apoptotic network

We first consider the miR-125 family of miRNAs, which is highly conserved throughout diverse species from nematodes to humans. Members of this family, such as miR-125a, miR-125b, and miR-125b-2, have been validated to be downregulated, exhibiting disease-suppressing properties in many conditions as well as disease-promoting functions [61]. It turns out that miR-125b is identified by IntegraMiR to be significantly downregulated – see Table 1. It has been recently suggested that miR-125b is an important component of a TP53 (p53) tumor-suppressor network whereas significant negative correlation has been reported between miR-125b and TP53 [62], [63]. Moreover, it has been shown that the p53-upregulated modulator of apoptosis BBC3 (PUMA) and NOXA are direct targets in p53-mediated apoptosis localized to mitochondria [64].

**Figure 7:**
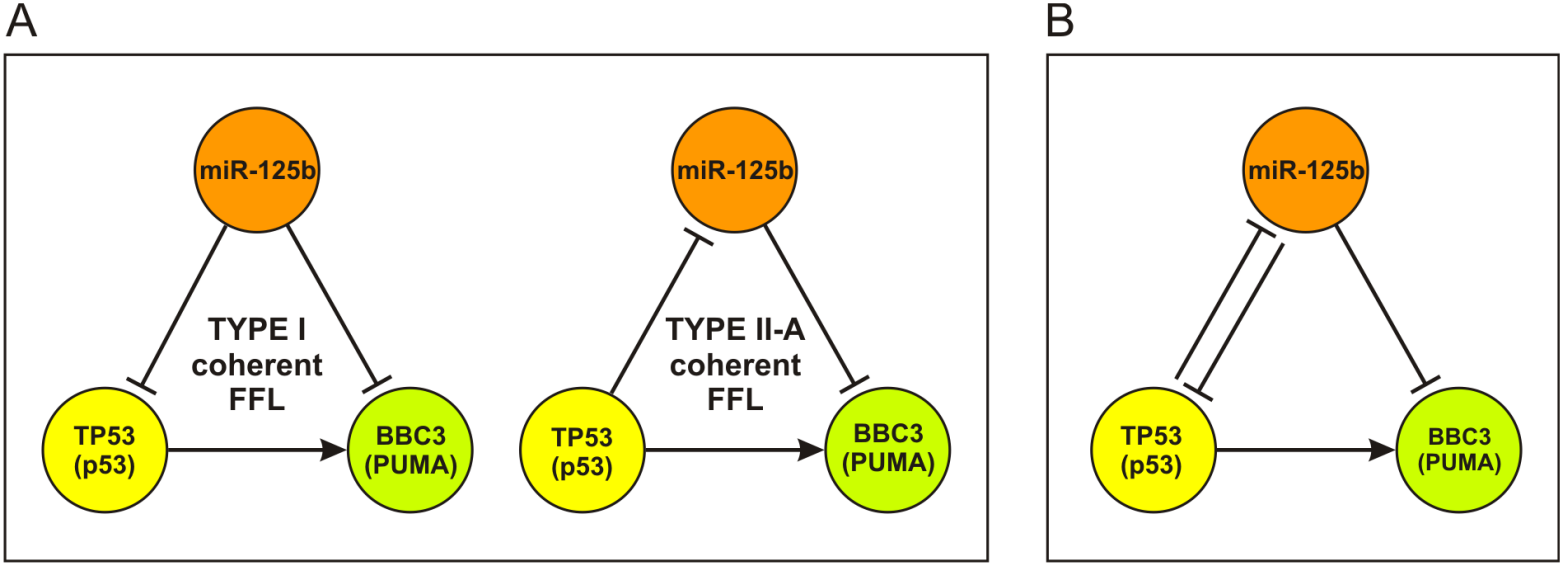
TP53 miRNA-mediated network model for apoptosis. IntegraMiR identifies two deregulated FFLs in PCa that model regulatory interactions among miR-125b, TP53 (p53), and BBC3 (PUMA). (A) Type I coherent and Type II-A coherent FFLs. (B) TP53 miRNA-mediated network model for apoptosis obtained by combining the two FFLs in (A).

To investigate systemic relations among these molecules of interest, we identified all deregulated FFLs predicted by IntegraMiR that contain miR-125b, TP53 (p53), BBC3 (PUMA) and NOXA. To focus our discussion on highly relevant FFLs, we consider only FFLs with nodes comprised of one of the four species of interest. We could not find FFLs that contain NOXA. However, we found one Type I coherent FFL and one Type II-A coherent FFL comprised of the other three species – see Figure 7A. Both FFLs are deemed by IntegraMiR to be deregulated in the prostate expression data.

The Type I coherent FFL suggests that miR-125b represses BBC3 while it reinforces this repression by targeting its transcriptional activator TP53. The Type II-A coherent FFL suggests that TP53 induces the transcription of BBC3 while it reinforces this induction by repressing miR-125b, an inhibitor of BBC3.

From a systemic point of view, if the Type I coherent FFL is functional in a specific condition in which miR-125b is significantly upregulated, we would expect the expressions of both TP53 and BBC3 to be repressed. As a consequence, miR-125b would assume an anti-apoptotic role in this setting. A similar argument can be made when miR-125b is significantly downregulated. As for the Type II-A coherent FFL, if TP53 is upregulated and active as a TF, we would expect miR-125b to be downregulated. As a consequence, BBC3 is expected to be significantly upregulated due to the concurrent upregulation of its transcriptional inducer, TP53, and the repression of its inhibitor, miR-125b. It is noteworthy that one cannot always expect to observe these exact relations in mRNA/miRNA expression data. It turns out that both FFLs depicted in Figure 7A are deregulated inconsistently based on the expression data.

The previous steps provide a fundamental understanding of the underlying structure of TP53 miRNA-mediated apoptotic network, which may not be directly attainable by looking at individual molecular interactions. In particular, by combining the two FFLs depicted in Figure 7A, we obtain the simple network depicted in Figure 7B. This network accentuates the mutual inhibition between miR-125b and the pro-apoptotic interaction between TP53 and BBC3, which is in line with the earlier reported observation of significant negative correlation between miR-125b and TP53 [62], [63]. The underlying double negative feedback means that upregulation of miR-125b will inhibit TP53 which will derepress miR-125b, a situation that can lead to the repression of BBC3. On the other hand, downregulation of miR-125b will derepress TP53 which will further repress miR-125b, a situation that may lead to significant activation of BBC3 and thus apoptosis. Double negative feedback loops are known to act as toggle switches that lead to different cell fates [65]. Interestingly, both TP53 and BBC3 have been validated to be targets of miR-125b according to miRTarBase. Moreover, the Type I FFL discussed above has been recently reported in [66], thus demonstrating the validity of the previous IntegraMiR predictions.

#### MYC-E2F1 miRNA-mediated cell proliferation network

**Figure 8:**
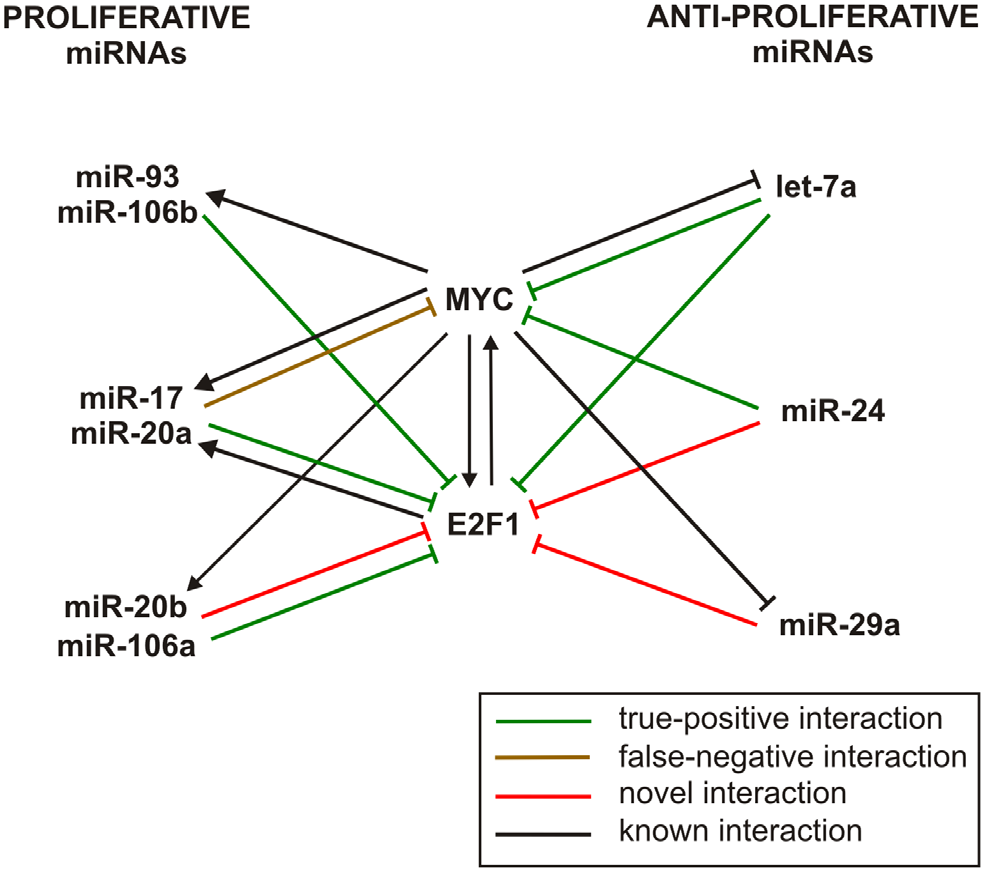
MYC-E2F1 miRNA-mediated network model for cell proliferation. A network of proliferative and anti-proliferative miRNAs interacting with MYC and E2F1 predicted by IntegraMiR. This network consists of 18 distinct FFLs: 8 Type I coherent, 2 Type II-A coherent, and 8 Type II-A incoherent. Green edges depict true-positive miRNA-target interactions identified by the predictive module of IntegraMiR, the brown edge predicts a false-negative miRNA-target interaction, red edges depict novel miRNA-target interactions, and black edges represent known interactions.

It is well known that deregulated expression or malfunction of the transcription factor MYC is one of the most common abnormalities in human cancers. Moreover, E2F1 is a member of the E2F family of TFs which are critical regulators of cell cycle and apoptosis. This TF regulates MYC and is transcriptionally targeted by MYC. Considering the fact that the miR-17/92 cluster and its paralogs have recently been shown to be tightly linked to the functions of MYC and E2F1 in the regulatory circuitry that controls cell proliferation [52], [54], [67], [68], we decided to identify all miRNA regulators predicted by IntegraMiR to interact with these critical TFs. This allowed us to delineate the regulatory network depicted in Figure 8, which we constructed from 18 distinct FFLs: 8 Type I coherent, 2 Type II-A coherent, and 8 Type II-A incoherent. A total of 9 miRNAs were predicted to interact both with MYC and E2F1, with 8 of the miRNA-target interactions being identified by the predictive module of IntegraMiR as being true-positives, 2 being identified as false-negatives, and 3 being novel predictions that need to be experimentally validated.

From a mechanistic point of view, the negative feedback loops and incoherent FFLs on the left-hand-side of Figure 8 ensure a tightly controlled regulation of cell proliferation. It has been argued in [53] that high levels of E2F proteins, especially E2F1, can induce apoptosis, and the negative feedback with miR-17 and miR-20a may dampen E2F activity following a physiologic proliferative signal, thereby promoting cell division rather than cellular death. On the other hand, the double-negative feedback loops and coherent FFLs on the right-hand-side of Figure 8 suggest anti-proliferative roles for the corresponding miRNAs, since these interactions repress MYC/E2F1 induced proliferation. As we mentioned before in our discussion related to Figure 5B, miR-24 and miR-29a exhibit tumor-suppressor roles, which is compatible with the network depicted in Figure 8. The miRNA let-7a has also been given a tumor-suppressor role in PCa [69], as well as in lung and renal cancers [70], [71].

### IntegraMiR provides further evidence of tumor-suppressor roles for miR-24, miR-29a and miR-145 in PCa

IntegraMiR identifies a large number of deregulated miRNA-target interactions in the four pathways we consider in this paper: 906 interactions in the TFG-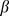 Signaling Pathway, 1,610 interactions in the Wnt Signaling Pathway, 1,017 interactions in the Prostate Cancer Pathway, and 895 interactions in the Adherens Junction Pathway – see Table S11. These pairs can potentially be used to form Type III regulatory loops.

To illustrate the functional scope and relevance of these interactions, we focus on the top three miRNAs depicted in Figure 4B found by IntegraMiR to be significantly downregulated. These are the tumor suppressor miRNAs miR-24, miR-29a, and miR-145 studied in Figure 5B. Using these miRNAs, we considered the deregulated miRNA-target interactions predicted by IntegraMiR and identified, as an example, those interactions relevant to the KEGG Prostate Cancer Pathway. IntegraMiR predicts a considerable number of deregulated interactions (45 for miR-24, 41 for miR-29a, and 40 for miR-145) with many common targets in this pathway. This may further be used to support the collaborative, tumor-suppressor role of these miRNAs in PCa, despite the fact that their predicted, genome-wide co-targeting features are relatively not much pronounced – see Figure 5B.

**Figure 9:**
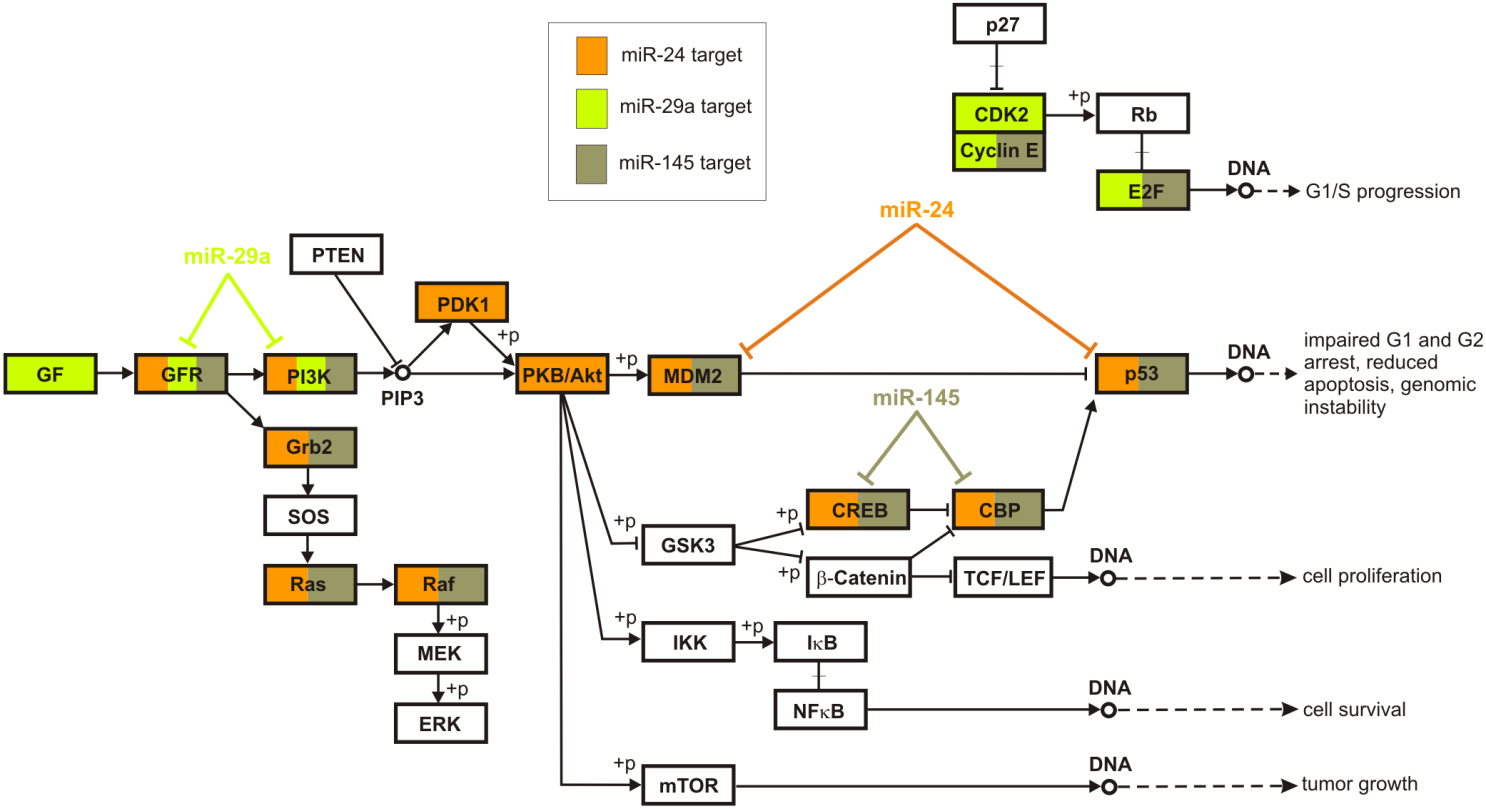
Predicted deregulated Type III regulatory loops in the Prostate Cancer Pathway. Portion of the Prostate Cancer Pathway, adopted from the KEGG database [39], [40], with the targets of miR-24, miR-29a and miR-145 that participate in deregulated Type III loops being color-coded. One example of a deregulated Type III loop is shown for each miRNA. All depicted Type III loops are novel and consistent, in the sense that the corresponding miRNA-target interactions are anti-correlated according to the data.

We also identified from Table S11 the consistently deregulated Type III regulatory loops associated with the three miRNAs, miR-24, miR-29a, and miR-145, in the KEGG Prostate Cancer Pathway by excluding the missing pathway interactions as well as interactions with indirect effects, as defined by the KEGG database. We depict the results in Figure 9. From all predicted interactions, only the interaction between miR-145 and IGF1R, a product of the GFR gene, as well as the interaction between miR-29a and PIK3R1, a product of the PI3K, are known (i.e., are true-positives). It turns out that several Type III loops predicted by IntegraMiR encompass genes that have established oncogenic roles, such as the genes in the PI3K-Akt backbone and the Ras and Raf genes in the MAPK signaling section of the pathway. This observation thus provides further support for the tumor-suppressor roles of these miRNAs in PCa.

### IntegraMiR Leads to a Novel Regulatory Circuit for Epithelial-to-Mesenchymal Transition (EMT)

EMT is a complex gene expression program characterized by loss of cell adhesion through repression of CDH1 (E-cadherin) and activation of genes associated with motility, invasion and stemness [72]. EMT is activated during embryonic development and adult tissue remodeling. In epithelium-derived tumors however, EMT seizes to promote metastasis and gain of stem cell phenotypes [50]. Since modulation of CDH1 expression levels is considered to be a major theme of epithelial plasticity, both in non-oncogenic and oncogenic EMT, we sought to construct and investigate an integrated circuit that controls EMT in PCa based on IntegraMiR predictions.

A natural approach towards this goal is to first identify the most relevant molecular species to build an initial network and subsequently expand this network with additional species. Since our main interest here is to determine FFLs mainly involved in pathological conditions related to EMT and since the most common biochemical change associated with EMT is loss of CDH1 expression, we decided to focus on CDH1 repressors and their corresponding regulatory network. CDH1 transcriptional repressors, such as SNAI1 (SNAIL), SNAI2 (SLUG), ZEB1, ZEB2 (SIP1), E12/E47, and TWIST have traditionally been implicated in promoting EMT in various systems of embryonic development and tumor progression [72], [73]. Among these repressors, we found that SNAI2 and ZEB1 are associated with FFLs predicted by IntegraMiR – see Tables S5–S10. It is important to note that the TGF-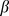 Signaling Pathway induces the transcription of SNAI2 (SLUG), which in turn activates ZEB1 [74], [75]. Furthermore, the miR-200 family of miRNAs (miR-200a, miR-200b, miR-200c, miR-141 and miR-429) has been shown to play a major role in EMT [72], [76]. Among the family members, miR-200b, miR-200c and miR-141 have been identified by IntegraMiR to be significantly deregulated in PCa – see Table 1.

**Figure 10:**
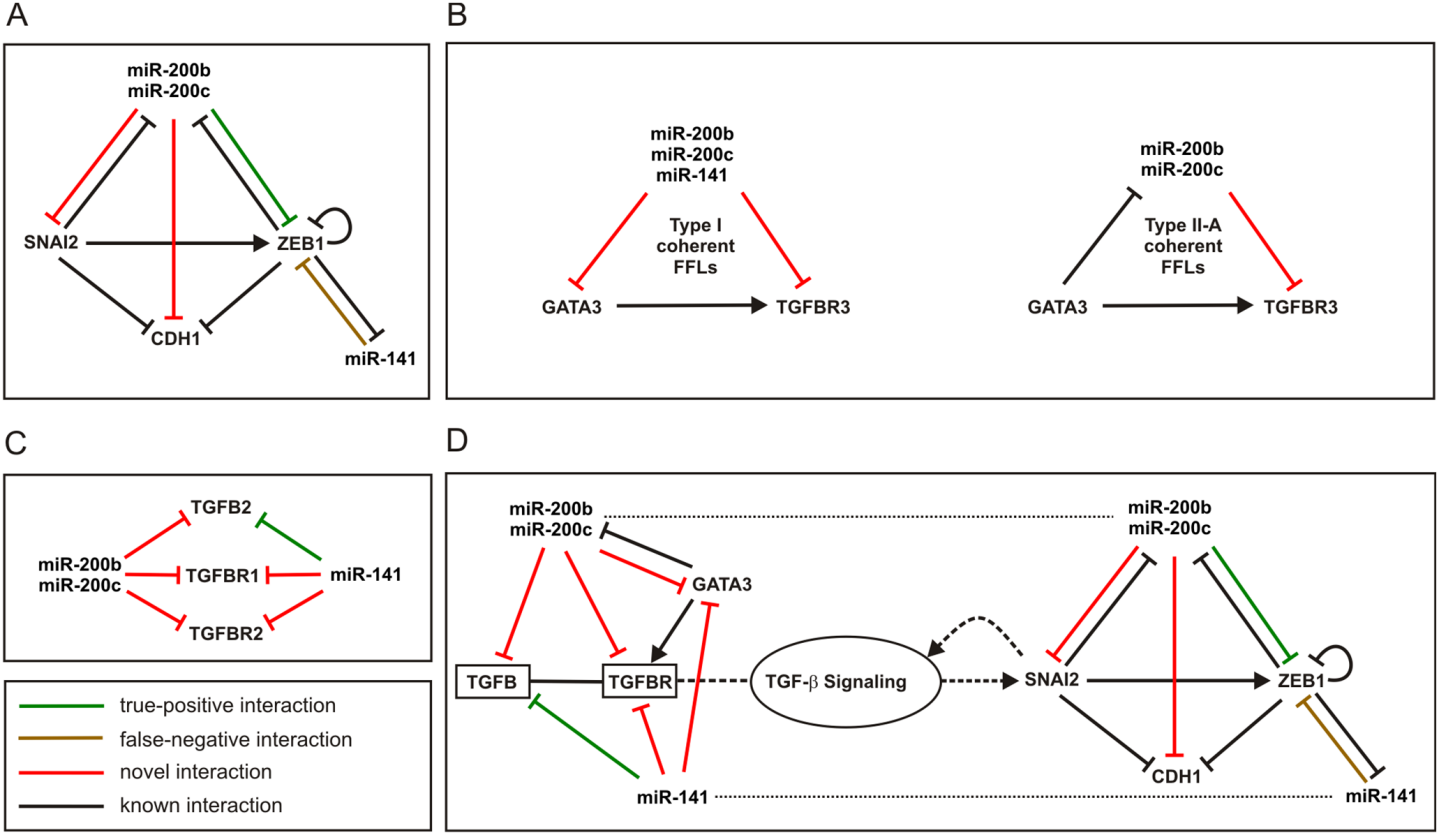
Predicted regulatory circuits controlling EMT. (A) An initial regulatory circuit, predicted by IntegraMiR, controlling EMT in PCa through regulation of CDH1 (E-cadherin) transcriptional repressors. This network consists of 14 distinct FFLs: 2 Type I coherent, 5 Type I incoherent, 2 Type II-A coherent, and 5 Type II-B incoherent. (B) The five FFLs predicted to be (consistently) deregulated in PCa by IntegraMiR comprising miR-200b, miR-200c, or miR-141, and GATA3 and TGFBR3. (C) The nine deregulated miRNA-target interactions involving miR-200b, miR-200c, and miR-141 as well as the TGFB ligands and receptors. (D) An extended integrated regulatory circuit, predicted by IntegraMiR, controlling EMT through TGF-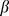 signaling and regulation of CDH1 transcriptional repressors. In these figures, green edges depict true-positive miRNA-target interactions identified by the predictive module of IntegraMiR, brown edges represent false-negative miRNA-target interactions, red edges depict novel miRNA-target interactions, and black edges depict known interactions.

To delineate a basic network for EMT regulation, we first single out all deregulated FFLs whose nodes comprise only entries among the molecular species we have identified: miR-200b, miR-200c, miR-141, CDH1, SNAI2, and ZEB1– see Figure S2. These FFLs are deemed to be consistently deregulated by IntegraMiR. For miR-141, we discovered two loops whereas for miR-200b and miR-200c, we discovered six loops for each miRNA with identical types. We then constructed the network depicted in Figure 10A by combining these FFLs.

To extend this basic network, we regard the fact that TGF-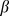 signaling induces the transcription of SNAI2 and consider the recent discovery that SNAI2 and TGF-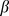 signaling interact in a positive feedback loop [73], [77]. We then hypothesized that we may observe a (mutually) inhibitory relation between members of the miR-200 family and upstream factors in TGF-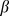 signaling due to the fact that these miRNAs interact with SNAI2 in a mutually inhibitory fashion, as predicted by the network depicted in Figure 10A. To constrain this investigation to a tractable number of transcripts, among the numerous transcripts associated with TGF-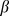 signaling, we focus on the very first elements of this pathway: three TGFB isoforms (TGFB1, TGFB2, TGFB3) and three TGFB receptors (TGFBR1, TGFBR2, TGFBR3).

We should note here that, among TGFB cell surface receptors, TGFBR3 has the most abundant expression and it shows the highest affinity for binding TGFB2 ligand among all three TGFB ligand isoforms. While TGFBR3 does not have a functional kinase domain to activate TGF-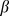 signaling, it helps TGFB ligands be presented to TGFBR2, which leads to the association and phosphorylation of TGFBR1 and subsequent activation of TGF-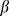 signaling by phosphorylation of Smad2 or Smad 3 proteins [78]. Reduced or loss of TGFBR3 expression has been observed in many types of cancer, such as prostate, pancreatic, breast, renal, and lung cancer [79]–[83].

We identified all FFLs predicted to be deregulated by IntegraMiR (see Tables S5–S10) comprising miR-200b, miR-200c, or miR-141, and one of the TGFB ligand isoforms or one of the TGFB receptors. This produced the three Type I coherent and the two Type II-A incoherent FFLs depicted in Figure 10B all of which are deemed to be consistently deregulated in the data. We also identified all deregulated miRNA-target interactions for miR-200b, miR-200c, and miR-141 associated with the KEGG TGF-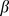 Signalling Pathway (see Table S11), with the target being one of the TGFB ligand isoforms or one of the TGFB receptors. We depict the results in Figure 10C, which shows that each of these miRNAs targets TGB2, TGFBR1, and TGFBR2. Among these interactions, only TGFB2 has been experimentally verified to be a target of miR-141 according to miRTarBase.

By incorporating the results depicted in Figures 10A–C, we obtain the extended circuit for EMT regulation depicted in Figure 10D. To simplify presentation, we lump specific interactions of the miRNAs with individual TGFB receptors in a single block. Interestingly, this circuit predicts a mutually inhibitory relation between miR-200b, miR-200c and GATA3, a recently discovered transcriptional activator for TGFBR3 [84]. Moreover, miR-200b, miR-200c, and miR-141 are predicted to repress the upstream TGFB2 ligand and receptors in a Type III regulatory loop. The resulting integrated regulatory circuit provides a hypothesis for a novel and more comprehensive model for regulation of EMT at the transcriptional, post-transcriptional and signaling levels, by means of miR-200 family members, TGF-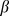 signaling and the corresponding transcriptional program.

### Transcriptional, Post-transcriptional and Signaling Deregulation, Coupled with Known Genetic and Epigenetic Alterations, Reveal a Relatively Comprehensive Model for PCa Development

**Figure 11:**
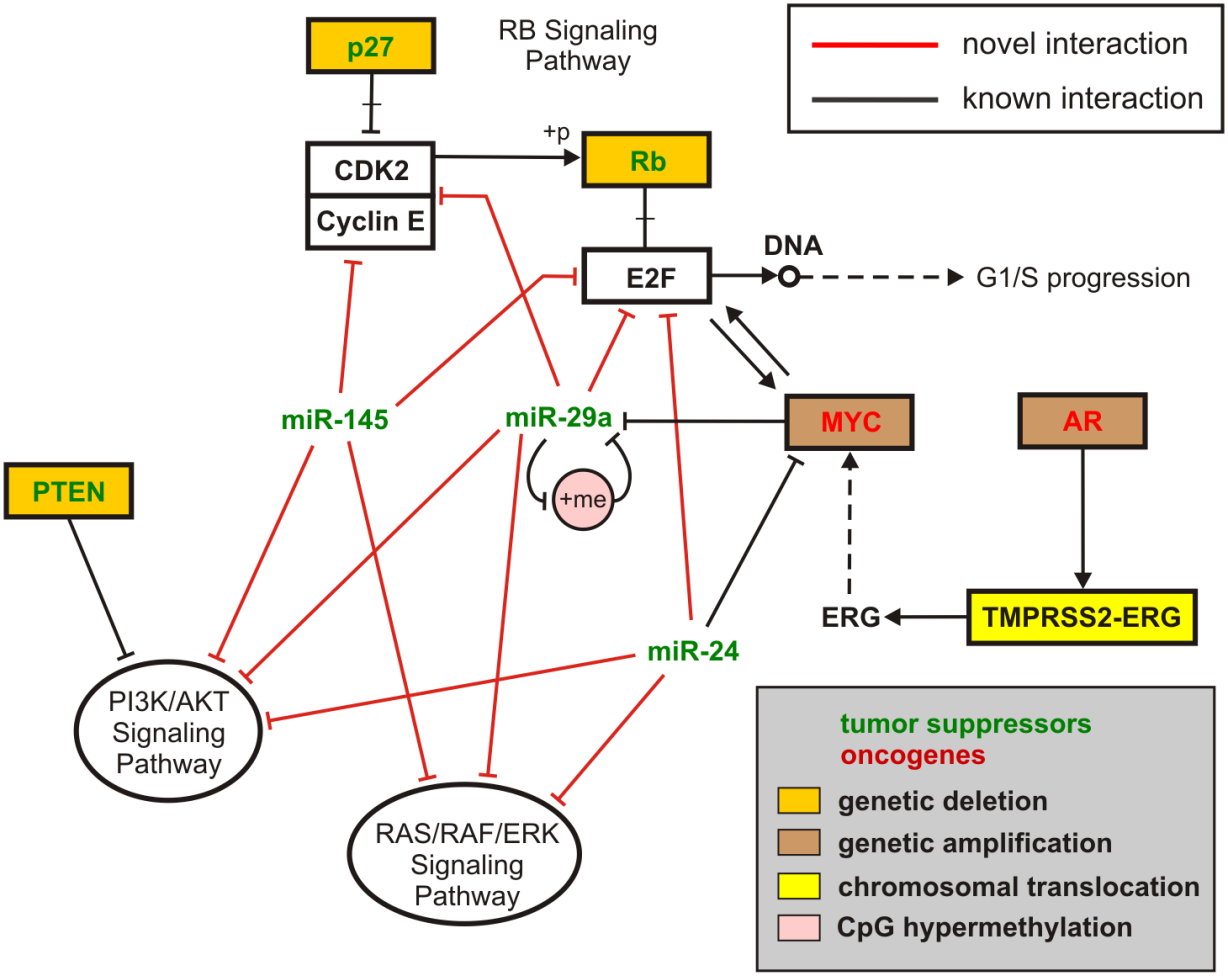
Integrative miRNA-mediated model for PCa development. A snapshot of a high-level integrative miRNA-mediated model for PCa development which encapsulates major sources of deregulation at the transcriptional, post-transcriptional, and signaling levels, coupled with genetic and epigenetic alterations.

To discern the effectiveness of the integrative analyses we carry out in this study, we combined information from the results depicted in Figures 8 & 9, as well as current knowledge of certain crucial genetic and epigenetic alterations in PCa (which we will be discussing shortly), to delineate the model depicted in Figure 11. This model encapsulates some major sources of deregulation in PCa at the transcriptional, post-transcriptional, signaling, and genetic/epigenetic levels, as opposed to models that only consider deregulation at just one level, which may not be capable of capturing the overall behavior of the underlying network. We use this model to discuss how genetic and epigenetic alterations could propagate in cellular regulatory networks through circuits identified in this study and, therefore, adversely affect gene regulation. These pieces of crucial information represent a relatively comprehensive model for PCa development.

It has been demonstrated that chromosomal translocation involving TMPRSS2 (PSA-regulated gene transmembrane protease, serine 2), an androgen receptor (AR)-regulated gene, and a member of the ETS family of TFs (predominantly ERG) is present in about half of all PCa cases [45]. This rearrangement in prostate cancer leads to androgenic induction of ERG expression (see Figure 11) and the critical outcomes associated with its overexpression in PCa [85]. In particular, it has been suggested that ERG overexpression in PCa may contribute to the neoplastic process by activating MYC and by abrogating prostate epithelial differentiation [86]. Moreover, global analysis of copy-number alterations (CNAs) in PCa has reported dramatic amplifications of oncogenes, such as MYC (on 8q24.21) and AR (Xq12), deletions of tumor suppressor genes, such as PTEN (10q23.31), RB1 (13q14.2), TP53 (17p31.1) and CDKN1B (due to the broader deletion of the 12p13.31-p12.3 genomic region), and interstitial 21q22.2–3 deletion spanning ERG and TMPRSS2 [87]. Finally, based on the integration of CNA, transcriptome and mutation data, it was found that PI3K, RAS/RAF and RB signaling were commonly altered in primary tumors and metastases [87]. Moreover, it was stated that the data provided strong rationale for exploring the clinical activity of PI3K pathway inhibitors.

Interestingly, the findings depicted in Figures 8 & 9 characterize miR-24, miR-29a, and miR-145, which are identified by IntegraMiR to be significantly downregulated, as inhibitors of the PI3K/AKT, RAS/RAF/ERK and RB signaling pathways through specific FFLs and Type III loops, as depicted in Figure 11, and suggest tumor suppressor roles for these miRNAs, coordinately cooperating with the tumor suppressors PTEN, CDKN1B (p27) and RB1 (Rb).

As a notable example, the Rb tumor suppressor gene product in Rb signaling is known to be a target of CDK2 (cyclin dependent kinase 2). When Rb is dephosphorylated, it interacts with E2F transcription factors and, in this way, prevents transcription of genes required for progression through the cell cycle. On the other hand, when Rb is phosphorylated by cell cycle dependent kinases, such as CDK2, it no longer interacts with E2F and the cell cycle proceeds through the G1-S checkpoint. The results depicted in Figure 11 identify miR-29a and miR-145 as potential inhibitors of the CDK2/Cyclin E complex and E2F through FFLs and Type III regulatory loops and suggest that these miRNAs work in concert with p27 and Rb tumor suppressors, preventing passage from the G1 to the S phase.

In addition to the previously discussed genetic alterations and their effect on gene regulation, recent studies have found that miRNAs are both regulated epigenetically and play roles in epigenetic regulation of protein coding genes in different types of cancer, including PCa [59], [88], [89]. A recently validated example, which is relevant to our discussion, is miR-29a. It was discovered in [59] that the promoter region of miR-29a harbors numerous CpG sites. Moreover, it was determined that the experimentally measured methylation index of the miR-29a promoter was higher in the PCa cell group than in the prostate epithelial cell group, resulting in significant downregulation of miR-29a expression in PCa. More interestingly, miR-29a has been shown to play tumor suppressor roles by reciprocally targeting DNA methyltransferases (DNMTs), which are key regulators of methylation of CpG islands [88]–[90].

We summarize these findings in the model depicted in Figure 11, in which the red edges represent novel interactions predicted by IntegraMiR. In particular, edges emanating from the three miRNAs that target the two signaling pathways at the bottom represent the novel miRNA interactions depicted in Figure 9. The resulting model suggests that upregulation of the oncogene MYC could take place due to genetic amplification and/or by ERG through TMPRSS2-ERG gene fusion. The upregulated MYC could then initiate a proliferative program, for instance, through the depicted MYC-E2F interaction, as well as by inhibiting the tumor suppressor miR-29a. In addition, other genetic and epigenetic alterations, for instance hypermethylation of miR-29a or deletion of PTEN, p27 and Rb, could further suppress the level of these tumor suppressor miRNAs and genes, leading to the activation of PI3K/AKT, RAS/RAF/ERK and RB signaling, and a consequent uncontrolled cellular growth.

It is important to emphasize at this point that miRNAs have attracted attention due to their diagnostic as well as therapeutic potential. Inactivating oncogenic miRNAs or restoring tumor suppressor miRNAs offers great prospects for cancer therapy [91]–[95]. As an important practical application, chromatin-modifying drugs, such as DNA methylation inhibitors, can be used to reactivate hypermethylated tumor suppressor miRNAs. Two DNMT inhibitors, 5-azacytidine and 5-aza-2′-deoxycytidine, have indeed been approved by the US Food and Drug Administration (FDA) for the treatment of myelodysplastic syndromes and acute myeloid leukemia [96].

## Discussion

The exquisite orchestration of molecular interactions in cells is essential for the normal homeostatic regulation of multicellular organisms. Systematic delineation of networks of such molecular interactions is a challenging task. Moreover, the identification of interaction networks deregulated in a particular disease may have profound effects on understanding the molecular causes that lead to the disease and may dramatically influence the development of effective strategies for pharmaceutical and therapeutic intervention.

Earlier computational tools have focused primarily on identifying pairwise miRNA-target and TF-target interactions, either by relying on sequence-based analysis or expression data [3]–[5]. As a consequence, they may produce an excessively large number of false-positive predictions making them inefficient for experimental follow-up.

More recently, two promising methods have been proposed to identify miRNA/TF interactions [26], [27], which are based on the hypothesis that certain regulatory circuits, defined as motifs [13], appear in a statistically over-represented manner in the human and mouse genomes [12]. However, and for a given motif structure (e.g., an FFL), these methods attempt to predict all interactions (the three edges of an FFL) by utilizing a narrow set of computational tools and limited biological information. Although the methods can be employed to provide insights into the prevalence of various motif instances in gene regulatory networks, the user must keep in mind that the results may contain a rather large number of possibly unreliable predictions for experimental validation due to the fact that these methods do not effectively utilize certain known biological information to appropriately constrain and systematically reduce the resulting predictions.

In this paper, we introduced IntegraMiR, a novel computational method for inferring deregulated miRNA/TF-mediated regulatory loops and networks that appear in a statistically over-represented manner in gene regulatory networks. IntegraMiR addresses the previous problems by appropriately constraining the statistical analysis of given mRNA/miRNA expression data and sequence-based target identification methods using relevant motif structures built by “prior” biological information readily available in existing databases. The main strength of IntegraMiR originates from its capacity to fuse information from multiple sources and incorporate several statistical techniques to exploit almost any accessible aspect of available information in the expression data to identify integrated regulatory loops and networks at the transcriptional, post-transcriptional and signaling levels. Therefore, IntegraMiR adds to the ongoing effort of developing effective computational techniques for network identification by utilizing available experimental data and existing biological knowledge in an effort to produce reliable predictions in a context-dependent manner.

To appropriately constrain the problem of predicting miRNA-target interactions, IntegraMiR focuses on specific types of three-node regulatory motifs and, in particular, FFLs that have attracted a great deal of attention in the literature. It is important to mention here that, in contrast to earlier work, such as that in [26], by identifying instances of deregulated FFL motifs and by using these motifs to construct interaction networks, IntegraMiR can also provide instances of two types of deregulated two-node motifs: miRNA-TF negative and double-negative feedback loops – see Figures 7B, 8, and 10D.

IntegraMiR identified a number of already validated and novel deregulated miRNA/TF-mediated interactions. Although our interest was focused on certain types of regulatory loops deregulated in PCa, the basic method can be easily modified to handle any other type of regulatory motif of interest and can be readily applied to other types of human disease, provided that appropriate miRNA and mRNA expression data are available. The examples considered in the “Results” section demonstrate that IntegraMiR is a powerful computational tool for miRNA/TF-mediated network prediction, which can effectively result in novel hypotheses for further experimental study and validation. We should point out that the output results produced by IntegraMiR, provided in the accompanying Tables S1–S12, can be used by interested investigators to formulate additional hypotheses for experimental validation, beyond the ones discussed in this paper, which are expected to lead to additional novel findings.

**Figure 12:**
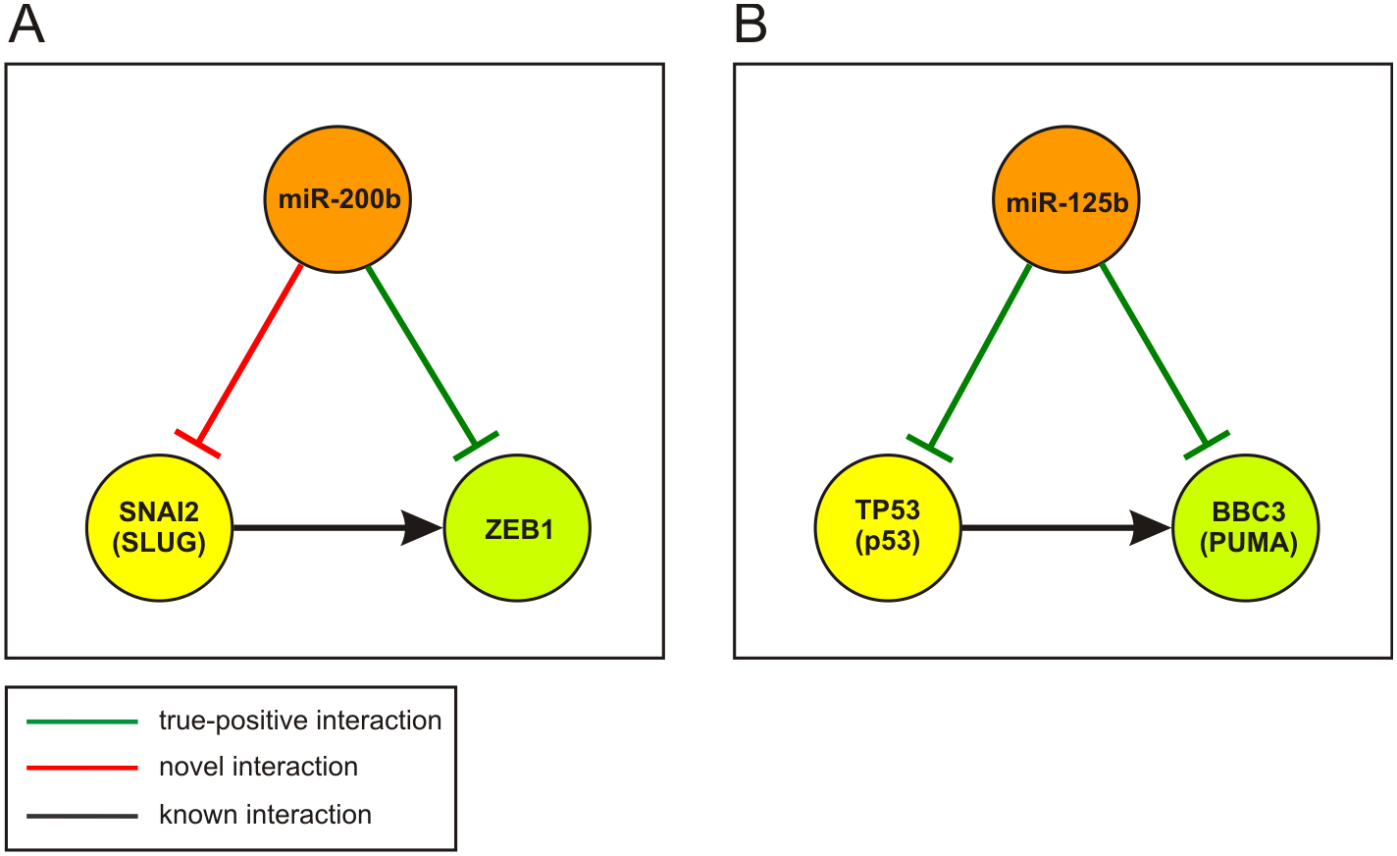
Examples of consistently and inconsistently deregulated FFLs identified by IntegraMiR. (A) A consistently deregulated Type I coherent FFL. (B) An inconsistently deregulated Type I coherent FFL. Green edges represent true-positive predictions, the red edge represents a novel prediction, and black edges represent known interactions. The red edges emanating from the miRNAs that target the two signaling pathways represent the novel interactions depicted in Figure 9.

IntegraMiR labels identified motifs into *consistent* and *inconsistent* loops, based on the rules depicted in Figure 3 (see also the “Materials and Methods” section). This is an additional piece of information that can be considered when evaluating the obtained results before carrying out experimental validation, when one seeks evidence based on expression data. As an illustrative example, we depict in Figure 12 two loops considered in the “Results” section – see Figure 7A and Figure S2. These are instances of a Type I coherent FFL, with the green edges representing true-positive predictions and the red edge representing a novel interaction. The FFL depicted in Figure 12A is identified by IntegraMiR to be consistently deregulated based on the data, whereas the FFL depicted in Figure 12B is identified to be deregulated inconsistently.

The consistency of the deregulated FFL depicted in Figure 12A implies that there is supporting evidence in the expression data to corroborate the intended reinforcing function modeled by this FFL. More specifically, when comparing tumor versus normal, the observed significant upregulation of miR-200b leads to significant downregulation of the transcription factor SNAI2 (SLUG) and to a consequent downregulation of ZEB1. On the other hand, the inconsistency of the deregulated FFL depicted in Figure 12B originates from the fact that, although the upstream inhibitor miR-125b is found by IntegraMiR to be significantly downregulated, and the opposite is true for the transcription factor TP53 (P53), the target gene BBC3 (PUMA) shows downregulation at the transcript level, which is contrary to the expected function modeled by this FFL.

Although all three interactions in an FFL, such as the one depicted in Figure 12B, may have been experimentally validated individually, we may still not be able to observe consistent deregulation among the FFL nodes at the transcript level. This situation may occur due to a number of biological or technical factors. For example, the known miRNA-target interactions available in miRTarBase may experimentally have been validated in certain cell type(s) and tissue(s) and may not take place in the context of interest (prostate tissue in our case). On the other hand, microarray experiments may not be able to capture the effect of translational repression by a miRNA (e.g., when this repression does not occur through mRNA degradation) or the fact that the mRNA level of a TF may not serve as a proxy for the corresponding protein-level activity. For example, in the case depicted in Figure 12B, although miR-125b is downregulated and the transcription factor TP53 transcript is upregulated based on the expression data, we may not have a high level of active TP53 protein in the nucleus that sufficiently correlates with the abundance of TP53 mRNA transcripts. As a result, the target BBC3 gene may not be transcribed in proportion to the level of the TP53 transcript. In addition to the above, each node in an FFL may not necessarily participate only in that specific FFL and there can be numerous FFLs identified for certain nodes. This means that, by focusing on just one FFL, we may not be able to capture the relevant overall behavior. To do so, we may have to consider all collaborating FFLs in concert, which could potentially provide a more accurate and comprehensive representation of gene regulation for a specific gene of interest (we did this in several settings discussed in the “Results” section). Finally, alternate effects due to mechanisms other than FFL regulation, such as alterations at the genetic and epigenetic levels, could give rise to behaviors and observations that cannot be modeled by FFLs.

**Figure 13:**
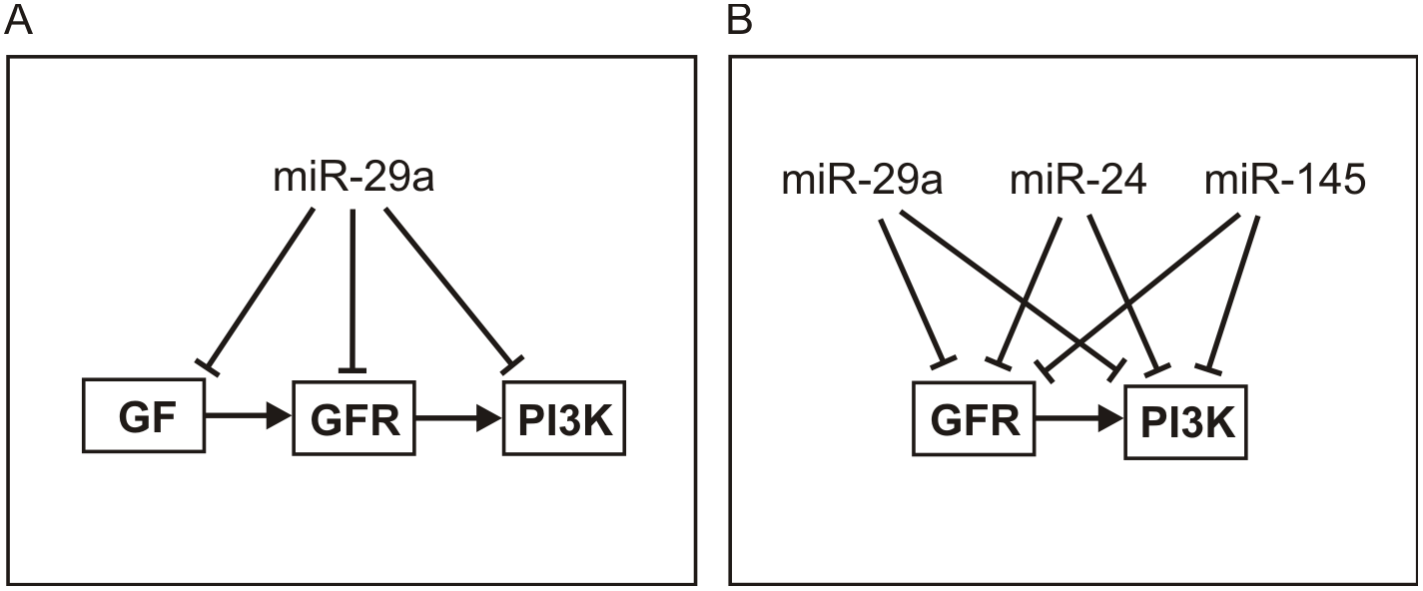
Complex regulatory motifs can be constructed from results obtained by IntegraMiR. (A) SIM motif of GF, GFR, and PI3K genes targeted by miR-29a in the KEGG prostate cancer pathway. (B) DOR motif of GFR and PI3K co-targeting by miR-29a, miR-24, and miR-145 in the KEGG prostate cancer pathway.

As we mentioned before, the two key hypotheses behind our interest in Type III loop motifs are that miRNAs play major roles in regulating signaling pathways due to their sharp dose-sensitive nature, and that targets of single miRNAs are more connected (i.e., interact) at the protein level than expected by chance. IntegraMiR identifies closely related miRNA targets on pathways deemed to be important in PCa and delineates certain miRNA-mediated three-node regulatory loops in the KEGG Prostate Cancer Pathway. As an example, we refer to the two consecutive Type III loops for miR-29a depicted in Figure 13A, which have been constructed from the results depicted in Figure 9. The obtained mechanism of a single miRNA regulating several closely related genes typically working together to perform a common task represents a single-input module (SIM) motif [13]. SIMs can partially explain how individual miRNAs could be potent regulators of pathway activity even though the effect of the miRNA on any single gene target may be modest [35], [97], [98].

It has also been demonstrated in [35] that targeting of a set of genes by multiple miRNAs could produce effects that are much more dramatic than the modest effects exerted by individual miRNAs. A notable example identified by IntegraMiR in the KEGG Prostate Cancer Pathway is the co-targeting of GFR and PI3K genes by miR-29a, miR-24 and miR-145 depicted in Figure 13B (which has been constructed from the results depicted in Figure 9). The resulting network structure represents a dense overlapping regulon (DOR) motif [13] in which several input miRNAs co-regulate a set of output genes (known as a regulon). Co-targeting in a DOR pattern presumably strengthens the notion that the miRNAs involved share similar regulatory roles. It is noteworthy that IntegraMiR can identify numerous examples of miRNA co-targeting in the context of FFLs as well – see Figure 8 and Figure S1. Clearly, the three-node loop motifs considered in this paper can serve as basic building blocks for identifying more complex regulatory motifs, such as SIMs and DORs [28], [65].

In principle, discoveries obtained by integrative computational approaches, similar to IntegraMiR, can provide systemic insights into the molecular biology of miRNA-mediated interactions and can, thereby, assign context-dependent biological functions to poorly understood roles of miRNAs. With further advances in genomics research, the need for integrative analysis approaches capable of utilizing information acquired from various sources is becoming more evident than ever before. It is through these findings that researchers can form hypotheses aimed at accurately dissecting context-dependent molecular mechanisms underlying physiological and pathological conditions of interest. Through these types of analyses, effective drug targeting and successful disease treatments will eventually be realized. MiRNAs pose promising potential in this context.

We finally conclude with some discussion on implementation issues.

IntegraMiR uses information from four databases, mSigDB, miRTarBase, TRANSFAC and TransmiR. If new and more informative databases become available in the future, information relevant to the problem discussed in this paper can be easily incorporated as part of the overall underlying strategy. For example, with the emergence and ever-increasing accessibility of high-resolution transcriptome data, by means of chromatin immunoprecipitation with sequencing (ChIP-Seq) experiments, together with regulation information, IntegraMiR could efficiently exploit such large-scale transcription factor-target information to obtain systems-level regulatory loops that could possibly account for much higher percentages in transcriptome changes.

We should note that a relatively large number of TF-target interactions are not included in the input to IntegraMiR owing to their unknown regulation type status in TRANSFAC and TransmiR. On the other hand, the method proposed in [26] does not utilize information on regulation type. As a result, although this method employs all TF-target interactions/associations available to it, it cannot be used to identify coherent/incoherent FFL subtypes, which is *the* information required to derive a systems-level understanding of regulatory networks. However, by using all available TF-target interactions regardless of their regulation type and by limiting their analysis to Type II FFLs, it was found in [26] that more than 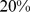 of transcriptome changes could be attributed to these FFLs. This result demonstrates that FFL-based analysis has the potential to explain a considerable percentage of transcriptome changes. Once additional information about regulation type is made available through future database updates, we expect that IntegraMiR will produce results that are capable of explaining a higher percentage of transcriptome changes, with systemic insights similar to the ones presented in this work, as opposed to the approach in [26].

In constructing FFLs, IntegraMiR considers loops comprising miRNA and TF nodes that are both significantly deregulated. The main reason for this choice is to focus primarily on FFLs that exhibit significant levels of deregulation at both regulator nodes, which could play a major role in explaining observed transcriptome changes. This is mainly because our confidence that an FFL contributes to transcriptome deregulation in PCa will be diminished if the upstream regulator is differentially expressed but the downstream regulator is not (or vice versa). Note that IntegraMiR can be easily adjusted to identify FFLs in which at least one regulator node is significantly deregulated. It is important however to understand that this adjustment, in combination with the high false-positive rate of sequence-based miRNA-target predictions, can result in an excessive number of predicted FFLs and relatively higher false-positive rates. This is due to the fact that this simple modification allows a combinatorially larger number of potential nodes to be considered by the method.

Finally, the ranking score obtained by employing Fisher’s method could be improved by using methods proposed to combine dependent statistical tests [99], [100]. However, due to lack of reliable between-node (and cross-platform) correlation estimation, accounting for dependencies is not feasible. Therefore, IntegraMiR uses Fisher’s method to indicate the significance for each FFL by a ranking score, rather than a *P* value. Upon availability of mRNA and miRNA expression data and techniques that could allow for reliable calculation of correlations, a possible future direction would be to incorporate such information into various aspects of the statistical analysis framework currently used by IntegraMiR to score FFLs more accurately.

## Materials and Methods

### Biological Samples

In this work, we use publicly available mRNA expression data obtained from a previously published study [101] involving normal and cancerous prostate tissue samples. The normal samples were acquired during radical prostatectomy from non-suspect (normal) peripheral areas of the prostate of 48 different individuals diagnosed with low-risk tumors. The cancerous samples were acquired from 47 patients diagnosed with high-risk tumors, before administering any medical treatment. Detailed discussion on the materials and methods used to obtain and prepare these samples can be found in [101]. We also use publicly available miRNA expression data from a previously published study [102] obtained from histologically confirmed *matched* malignant and peripheral nonmalignant prostate tissue samples extracted from 20 different patients with untreated prostate cancer (PCa). These samples were prepared from prostatectomy specimens using methods detailed in [102].

### Expression Profiling

In [101], the total RNA extracted from each normal and cancerous prostate tissue sample was used to produce mRNA expression profiles for 17,324 human mRNAs. This was done by mRNA microarray hybridization using the Affymetrix (Santa Clara, CA) GeneChip Whole Transcript Sense Target Labeling Assay in conjunction with Affymetrix 1.0 Human Exon ST microarrays. The MIAME-compliant mRNA microarray data can be found in the NCBI GEO database (www.ncbi.nlm.nih.gov/geo) with accession number GSE29079.

The tumor samples used to obtain the mRNA expression data were characterized by their disease subtype, based on their TMPRSS2-ERG gene fusion status, through a number of reliable assessments using ERG gene expression levels, nested RT-PCR, and quantitative PCR measurements [101], [103]. These data have also been validated with respect to an earlier study [87], which included matched miRNA expression data for a number of patients. Seventeen tumor samples were defined as TMPRSS2-ERG fusion-positive and twenty samples were defined as fusion-negative. The remaining ten tumor samples that could not be reliably categorized were labeled as unknown fusion status.

The miRNA profiling experiments performed in [102] used Affymetrix 1.0 GeneChip miRNA microarrays, whose content is derived from the miRBase miRNA database v11.0 (www.mirbase.org). These experiments produced expression data for 847 human miRNAs in *matched* normal and cancerous tissues. The data can be obtained from the NCBI GEO database using accession number GSE23022.

We should note here that several miRNA profiling studies have been published in the literature concerning PCa [104]–[109]. However, results on deregulation of particular miRNA genes have been highly inconsistent [110]. Seeking support for the reliability of the miRNA expression data used in the present study, we should mention that a major factor that possibly contributes to these inconsistencies is known to be variations in the miRNA expression data due, for example, to a different proportion of stromal cells in tissue preparation. The previous miRNA microarray experiments are based on micro-dissected tissue samples that avoid the previous issue. In addition, miRNA *in situ* hybridization experiments were run to evaluate the localization of miRNA-expressing cells and ensure that miRNA expression in tumor samples is indeed cancer cell-associated [102]. Moreover, the results were partially validated with RT-PCR and compared with a previous study on miRNA expression data from PCa tissue obtained by deep sequencing [49].

### Data Preprocessing

IntegraMiR analyzes the raw mRNA and miRNA expression data using the statistical computing environment R (www.cran.r-project.org). Both types of data are background-corrected and normalized using quantile normalization [111]. In addition, the method employs the robust multi-array average (RMA) as a measure of mRNA and miRNA expression levels [111].

### Multiple Hypothesis Testing/Surrogate Variable Analysis

Standard statistical tests used to identify differentially expressed genes between two conditions in a typical gene expression profiling study (as adopted by previous methods, e.g., see [26], [27]) become fundamentally flawed in the presence of unaccounted sources of variability (due to biological and experimental factors among others) [36]–[38]. As a consequence, many genes that are indeed differentially expressed in the data are not detected, whereas many others are falsely declared as positives [37], [112].

To address this problem and effectively exploit the molecular subtyping information in the available mRNA expression data, IntegraMiR incorporates surrogate variable analysis (SVA) [36], together with multiple hypothesis testing (MHT), to identify differentially expressed genes between two conditions. The method uses the Bioconductor (www.bioconductor.org) package SVA (written in R) to perform SVA in order to take into account biological variabilities and batch effects due to molecular subtypes categorized by TMPRSS2-ERG gene fusion status in the tumor samples. This step has been shown to improve biological accuracy and reproducibility in genome-wide expression studies and enhances the quality of subsequent statistical analysis [36], [37].

IntegraMiR applies MHT to test for the null hypothesis 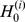: 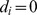 against the alternative hypothesis 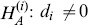, where
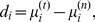
(1)with 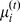 and 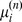 being the mean expression levels of the *i*-th transcript (mRNA or miRNA) in the tumorous and normal data, respectively. The Bioconductor package LIMMA (written in R), which implements a moderated t-statistic [48], is used on each data set to separately identify mRNAs and miRNAs that are differentially expressed between tumor and normal samples. Then, IntegraMiR applies the Benjamini-Hochberg method, described in [113], to control the false discovery rate (FDR) at 0.05. These steps produce two separate lists, 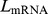 and 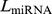, each containing 17,324 mRNAs and 847 miRNAs, with the corresponding FDR-adjusted *P* (or simply FDR) values and the direction of deregulation (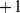 for upregulation and –1 for downregulation), as determined by the sign of the moderated t-statistic – see Table S13. The mRNAs and miRNAs with FDR 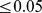 are considered as being differentially expressed between tumor and normal samples.

### Gene Set Enrichment Analysis

To further evaluate the statistical significance of certain mRNA and miRNA transcripts deemed not to be differentially expressed by MHT, IntegraMiR uses LIMMA to perform gene set enrichment analysis (GSEA), taking into account known biological knowledge about these transcripts – see [41]. Specifically, by employing the molecular signatures database mSigDB v3.1 (www.broadinstitute.org/gsea/msigdb), the method uses GSEA to evaluate the significance of non-differentially expressed TFs in 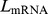 (MHT-based FDR 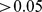) for which the target gene sets can be obtained from mSigDB. IntegraMiR forms gene sets indexed by these TFs, with the elements of each gene set being those mRNAs in 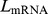 whose expressions are *directly* regulated by the indexing TF, as determined by mSigDB. It then uses GSEA to evaluate the statistical significance of each gene set to be enriched for deregulation, by using the default Wilcoxon rank-sum test. To adjust for multiple testing, IntegraMiR uses again the Benjamini-Hochberg method to control the FDR at 0.25– see [41]. This step produces a list 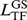 of TFs with the corresponding FDR values computed by MHT and GSEA – see Table S13. Only TFs with significantly enriched gene sets (GSEA-based FDR 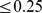) are included in this list. By combining lists 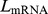 and 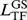, IntegraMiR finally forms a list 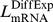 of mRNAs deemed to be differentially expressed by MHT or GSEA.

Likewise, IntegraMiR could use GSEA to further evaluate the statistical significance of non-differentially expressed miRNAs in 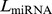 for which it is able to obtain their targets from the experimentally verified database miRTarBase v3.5 (http://mirtarbase.mbc.nctu.edu.tw – see [114]). Unfortunately, the limited number of experimentally validated miRNA targets available in miRTarBase is a restricting factor in constructing appropriate and sufficiently large gene sets in order to reduce the resulting bias (e.g., due to small gene set size or experimental predilection – see [115] for a discussion on this issue). Due to bias and relatively small gene set sizes, GSEA produces an appreciable number of significantly enriched gene sets for miRNAs that are not detected to be differentially expressed by MHT (FDR 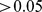), a majority of which are expected to be false positives. A possible way to remedy this situation is to improve the statistical power of GSEA by constructing sufficiently large gene sets of miRNA targets that have been validated to be important in PCa by at least one reliable experimental procedure (see [115] for a discussion). For this reason, IntegraMiR limits this step to a list 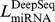 of 33 miRNAs that have been deemed to be significantly deregulated in PCa tissue using deep sequencing analysis [49]. Only gene sets having a minimum of *eight* elements are considered, as suggested in [116]. We should note here that it is not necessary to deal with this problem in the previous (and subsequent) application of GSEA, since all gene sets considered include a large and rather diverse number of elements in both cases.

To proceed, IntegraMiR uses miRTarBase to form gene sets indexed by miRNAs in 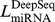, with MHT-based FDR values 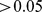 and with the elements of each gene set being the mRNA targets in 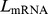 of the indexing miRNA, as determined by miRTarBase. It then uses GSEA to evaluate the statistical significance of a particular gene set enriched for an inverse direction of deregulation with that of the miRNA. The reason IntegraMiR uses an inverse relation is because many experiments used in the past to identify miRNA targets, with their results recorded in miRTarbase, have traditionally focused on observing an inverse relation between the expression level of a miRNA and its experimentally validated target(s). This step produces a list 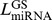 of experimentally validated (by deep sequencing analysis) miRNAs with the corresponding FDR values computed by MHT and GSEA – see Table S13. Only miRNAs with significantly enriched gene sets (GSEA-based FDR 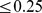) are included in this list. Finally, by combining lists 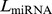 and 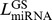, IntegraMiR forms a list 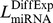 of miRNAs deemed to be differentially expressed by MHT or GSEA.

IntegraMiR also forms gene sets indexed by a specific KEGG signaling pathway included in mSigDB. The elements of each gene set are those mRNAs in 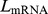 determined by mSigDB to be in the indexing pathway. The method then uses GSEA to evaluate the statistical significance of a particular gene set to be enriched for deregulation in the available mRNA data. This step produces a list 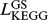 of gene sets, together with the associated KEGG signaling pathways and the corresponding GSEA-based FDR values – see Table S13. Only KEGG signaling pathways with significantly enriched gene sets (FDR 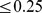) are included in the list.

We should point out here that mSigDB provides miRNA target gene sets as well. However, using GSEA to evaluate the statistical significance of these gene sets to be enriched for deregulation produces poor results. We believe that this is due to the possibility that many miRNA target gene sets provided by mSigDB are false positives. As a consequence, GSEA cannot produce meaningful statistical significance for these gene sets. As a consequence, IntegraMiR applies GSEA only on experimentally validated miRNA target gene sets in order to infer their statistical significance and complement the statistical analysis performed by simply using the available miRNA expression data.

### Target Identification

Since the goal of IntegraMiR is to delineate deregulated miRNA/TF-mediated gene regulatory loops from evidence provided in available data, the method focuses on loops containing differentially expressed miRNAs and TFs (based on their individual expression levels – via MHT analysis – or through their target interactions – via GSEA analysis). Note however that the target mRNAs associated with the loops of interest may not necessarily be differentially expressed. This is due to the fact that differential expression of a TF may not imply differential expression of the targeting mRNA (a TF may produce insignificant regulation of transcription), whereas miRNA targeting may result in direct translational repression without affecting mRNA abundance. Moreover, simultaneous differential expression of the miRNA and TF nodes of an incoherent Type I or Type II FFL may result in no deregulation of the associated mRNA since, in this case, downregulation (upregulation) of mRNA abundance by miRNA targeting may be counterbalanced by upregulation (downregulation) of transcription.

By following the previous rules, and for each differentially expressed TF in 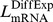, IntegraMiR uses information available in TRANSFAC v7.0 (public) (www.gene-regulation.com/pub/databases.html – see [117]) to identify the *directly* regulated genes in 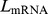 as well as to determine the regulation type (activation or repression). To access this information and provide the input to IntegraMiR, we first obtained for each TF its TRANSFAC-compatible annotation using the automated sequence annotation pipeline (ASAP) system [118], [119]. This process yields a list 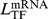 containing differentially expressed TFs in 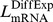, their gene targets in 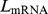, and the regulation type (activation or repression) for each target gene – see Table S13. TFs not predicted to target any mRNAs in 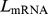 are not included in the list.

Likewise, IntegraMiR uses TransmiR v1.2 (http://202.38.126.151/hmdd/mirna/tf – see [120]) to identify differentially expressed TFs in 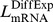 that *directly* regulate the transcription of miRNAs in 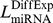. This produces a list 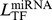 containing TFs from 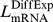, their corresponding transcriptional miRNA targets in 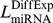, and the regulation type (activation or repression) for each targeted miRNA – see Table S13. TFs not predicted to target any miRNAs in 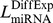 are not included in the list.

Finally, for each miRNA in 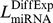, IntegraMiR performs sequence-based target prediction using miRecords (http://mirecords.umn.edu/miRecords – see [121]) with the filtering parameter set equal to 2. As a consequence, targets for each miRNA are predicted by at least two (out of eleven) different sequence-based target prediction algorithms incorporated in miRecords. Moreover, for each differentially expressed miRNA with experimentally validated target information in miRTarBase, we identified those mRNA targets not predicted by miRecords. This produced a list 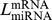 of differentially expressed miRNAs in 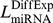 with the corresponding sequence-based target predictions in 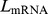 amended with (experimentally validated) targets from miRTarBase – see Table S13. miRNAs not predicted to target any mRNAs in 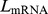 are not included in this list.

The reason we decided to use predictions by at least two different algorithms was to strike a balance between the number of false-positive and false-negative predictions. By setting the filtering parameter equal to 1, we obtain a large number of predictions (most of which are presumably false-positives) whereas by setting the filtering parameter equal to 3, we obtain a very small number of predictions (which presumably indicates a large number of false-negatives for the prediction). Note finally that miRecords provides a database for experimentally validated miRNA targets as well, but we decided to use miRTarBase instead, since the latter database is up-to-date, unlike the former which dates back to November 2010.

### Construction of Regulatory Loops

IntegraMiR constructs Type I FFLs by first identifying (TF, mRNA) pairs using the list 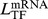. It then forms triplets (miRNA, TF, mRNA), such that a miRNA simultaneously targets the TF and the mRNA, as determined by the list 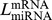
– see Figure 1. Likewise, IntegraMiR constructs Type II FFLs by first identifying (TF, miRNA) pairs from the list 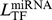. It then forms triplets (miRNA, TF, mRNA), such that the mRNA is directly regulated by the TF and is simultaneously targeted by the miRNA, as determined by the lists 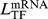 and 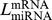, respectively. The method finally delineates all miRNA-target interactions in the four deregulated KEGG pathways under consideration (TGF-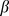 Signaling, Wnt Signaling, Prostate Cancer, and Adherens Junction) by first looking into the gene sets associated with each pathway (obtained from the KEGG database), by filtering out the genes that are not expressed in the data, and by identifying the targets of each miRNA as determined by the list 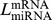. In addition, IntegraMiR constructs Type III loops by taking gene pairs (G-1, G-2) such that their corresponding proteins could potentially interact with each other according to the pathway map provided by KEGG database. It then highlights triplets (miRNA, G-1, G-2) such that the miRNA is predicted to target at least one transcript from each gene, as determined by the list 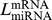. We carried out this step to identify, as an example, Type III loops in the KEGG Prostate Cancer Pathway for certain miRNAs.

Each edge depicted in Figure 1 connecting a miRNA with its target is naturally repressing. The list 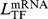 provides the regulation type (activation or repression) for each edge connecting a TF with a mRNA whereas the list 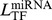 provides the regulation type (activation or repression) for each edge connecting a TF with a miRNA.

### Significance Ranking of FFLs

For each constructed FFL, IntegraMiR calculates its statistical significance by employing the following procedure. First, by using the lists 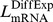, 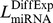, and 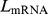, it associates with each node of the FFL a binary value (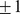), which indicates the direction of deregulation of the node. Moreover, it assigns the MHT-based FDR value corresponding to the particular transcript (TF, mRNA, or miRNA) represented by the node, which quantifies the significance of the transcript’s deregulation. To evaluate the statistical significance of each FFL, IntegraMiR first assumes that the FFL is not deregulated if each one of its nodes 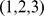 is not deregulated. It then constructs a hypothesis testing procedure to test for the null hypothesis 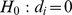, for every *i*, where 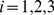, against the alternative hypothesis 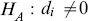, for at least one *i*, where 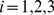, with 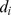 given by Eq. (1), with 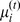 and 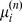 being the mean expression levels of the transcript (TF, mRNA, or miRNA) assigned at node *i* of the FFL in the tumorous and normal data, respectively. To do so, IntegraMiR uses Fisher’s method [44], [122] based on the summary test statistic.


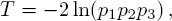
(2)

where *p*_1_, *p*_2_, and *p*_3_ are the *P* values obtained by MHT for nodes 1, 2, and 3, respectively. Under the null hypothesis, each (non-adjusted) *P* value obtained by MHT will have a uniform distribution between 0 and 1. Assuming that these values are obtained from independent statistical tests, the statistic *T* follows a chi-square distribution with *six* degrees of freedom, from which a combined value is obtained that is used to score each FFL.

We should note that these statistical tests depend on each other in general. It turns out that Fisher’s method may result in a combined *P* value that will be smaller than the *P* value which could be obtained if dependencies among the statistical tests used could be appropriately taken into account. For this reason, we regard Fisher’s method as producing a *score* for each FFL and not a formal *P* value [123]. As a consequence, we expect that IntegraMiR will produce a larger set of deregulated FFLs than a (hypothetical) hypothesis testing method that properly considers the underlying dependence of the individual tests. In the absence of any prior information however, accounting for these dependencies is a difficult task [99], [100], which we cannot satisfactorily address in this paper.

### Consistent Regulatory Loops

Since the functional roles of the FFLs considered in this paper are different, IntegraMiR groups them into five distinct categories: Type I coherent, Type I incoherent, Type II coherent, Type II incoherent, and Type III – see Figure 1. In addition, the method sorts Type II FFLs into two distinct subgroups, Type II-A and Type II-B, and marks as “consistent” those loops discovered to be deregulated in a manner compatible with the underlying edge structure. To do so, note that molecular species joined by an activating edge are expected to exhibit *correlated* deregulation whereas species joined by a repressing edge are expected to exhibit *anti-correlated* deregulation. Taking this fact into account, IntegraMiR marks deregulated loops as being *consistent* by using the rules depicted in Figure 3. For example, a deregulated Type I coherent FFL is said to be consistent if it comprises an upregulated miRNA node and downregulated TF and mRNA nodes, or a downregulated miRNA node and upregulated TF and mRNA nodes. A deregulated FFL that does not follow these rules is said to be *inconsistent*.

### Extracting Regulatory Loops

The results obtained by IntegraMiR, tabulated in the Tables S5–S10, contain a large number of deregulated Type I and Type II FFLs. To identify deregulated FFLs for specific miRNAs, TFs, or genes, we must search these results and extract those FFLs that contain the molecular species of interest. Moreover, identifying deregulated Type III loops for specific pairs of genes, requires construction of such loops from the results tabulated in Table S11. Extracting regulatory loops from the results can be done automatically by using two programs, FFLS and LOOPS, written in R. More details on these programs and the associated code can be freely downloaded from www.cis.jhu.edu/~goutsias/CSS%20lab/software.html. We have used these programs to obtain the results depicted in Figures 7, 8, 10, 12 and 13.

## Supporting Information

Figure S1 **In PCa, IntegraMiR predicts consistent deregulation of Type II-B coherent FFLs, comprising 6 miRNAs from the miR-17 family, which are activated by the oncogenic transcription factor MYC, and 33 mRNAs in the set on the left-hand-side.** It also predicts consistent deregulation of Type II-B incoherent FFLs comprising the same miRNAs and MYC and 46 mRNAs in the set on the right-hand-side.
(TIF)

Figure S2 **Deregulated FFLs predicted by IntegraMiR with nodes comprising only entries among miR-200b, miR-200c, and miR-141, as well as CDH1, SNAI2 (SLUG), and ZEB1.** The FFLs are consistently deregulated based on the data. Green edges depict true-positive miRNA-target interactions identified by the predictive module of IntegraMiR, brown edges represent false-negative miRNA-target interactions, red edges depict novel miRNA-target interactions, and black edges depict known miRNA-target interactions.
(TIF)

Table S1
**Differentially expressed mRNAs, obtained by MHT, with FDR-adjusted** ***P*** **values 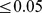.**
(XLSX)

Table S2
**Differentially expressed mRNAs, obtained by MHT, with FDR-adjusted** ***P*** **values 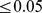 and fold changes 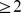, for overexpressed mRNAs, and 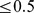, for repressed mRNAs.**
(XLSX)

Table S3
**Differentially expressed miRNAs, obtained by MHT, with FDR-adjusted** ***P*** **values 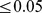.**
(XLSX)

Table S4
**Differentially expressed mRNAs (TFs), obtained by GSEA, with MHT FDR-adjusted** ***P*** **values 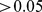 and GSEA FDR-adjusted** ***P*** **values 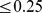.**
(XLSX)

Table S5 **Deregulated Type I coherent FFLs, predicted by IntegraMiR.**
(XLSX)

Table S6 **Deregulated Type I incoherent FFLs, predicted by IntegraMiR.**
(XLSX)

Table S7 **Deregulated Type II-A coherent FFLs, predicted by IntegraMiR.**
(XLSX)

Table S8 **Deregulated Type II-A incoherent FFLs, predicted by IntegraMiR.**
(XLSX)

Table S9 **Deregulated Type II-B coherent FFLs, predicted by IntegraMiR.**
(XLSX)

Table S10 **Deregulated Type II-B incoherent FFLs, predicted by IntegraMiR.**
(XLSX)

Table S11
**Deregulated miRNA-target interactions, associated with the TGF-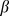, Wnt, Prostate Cancer, and Adherens Junction KEGG Pathways, which can be potentially used to construct Type III loops.**
(XLSX)

Table S12 **Co-targeting miRNA-TF pairs, extracted from the deregulated FFLs in Tables S5–S10, categorized by their interaction type.**
(XLSX)

Table S13 **Lists of mRNAs, TFs, miRNAs, and their targets used to construct deregulated loops and rank their statistical significance.**
(PDF)
