# Supplementary figures and images for: Integrative Identification of Deregulated MiRNA/TF-Mediated Gene Regulatory Loops and Networks in Prostate Cancer
Source: PLoS One. 2014 Jun 26;9(6):e100806. doi: 10.1371/journal.pone.0100806 (PMC4072696; doi:10.1371/journal.pone.0100806)

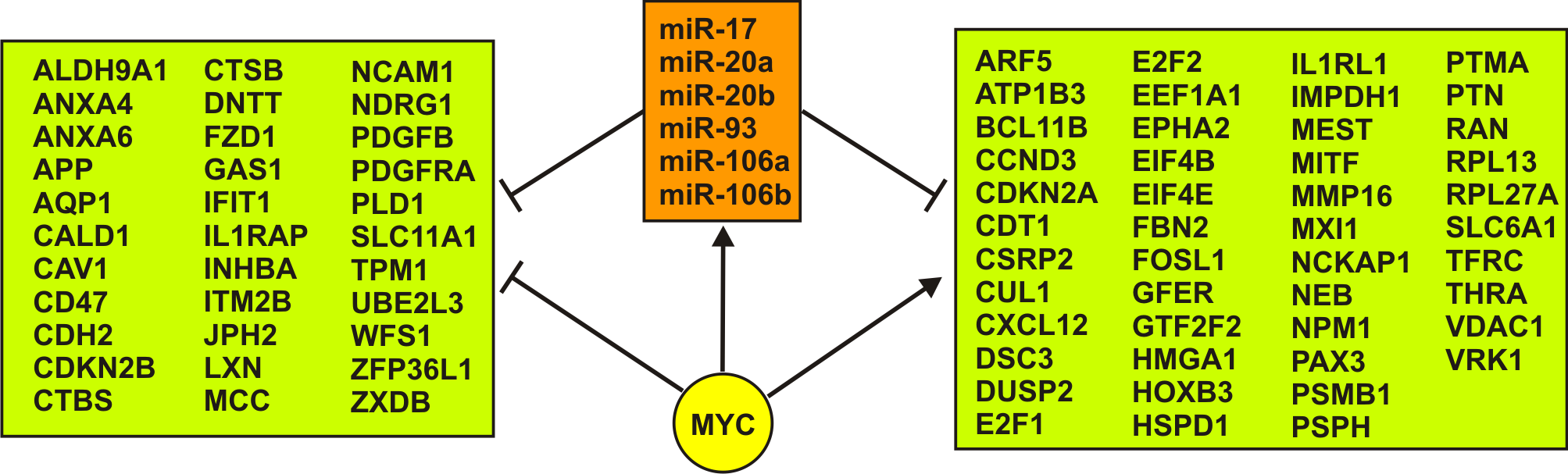

Supplement: Figure S1 — In PCa, IntegraMiR predicts consistent deregulation of Type II-B coherent FFLs, comprising 6 miRNAs from the miR-17 family, which are activated by the oncogenic transcription factor MYC, and 33 mRNAs in the set on the left-hand-side. It also predicts consistent deregulation of Type II-B incoherent FFLs comprising the same miRNAs and MYC and 46 mRNAs in the set on the right-hand-side. (TIF) [file pone.0100806.s001.tif]

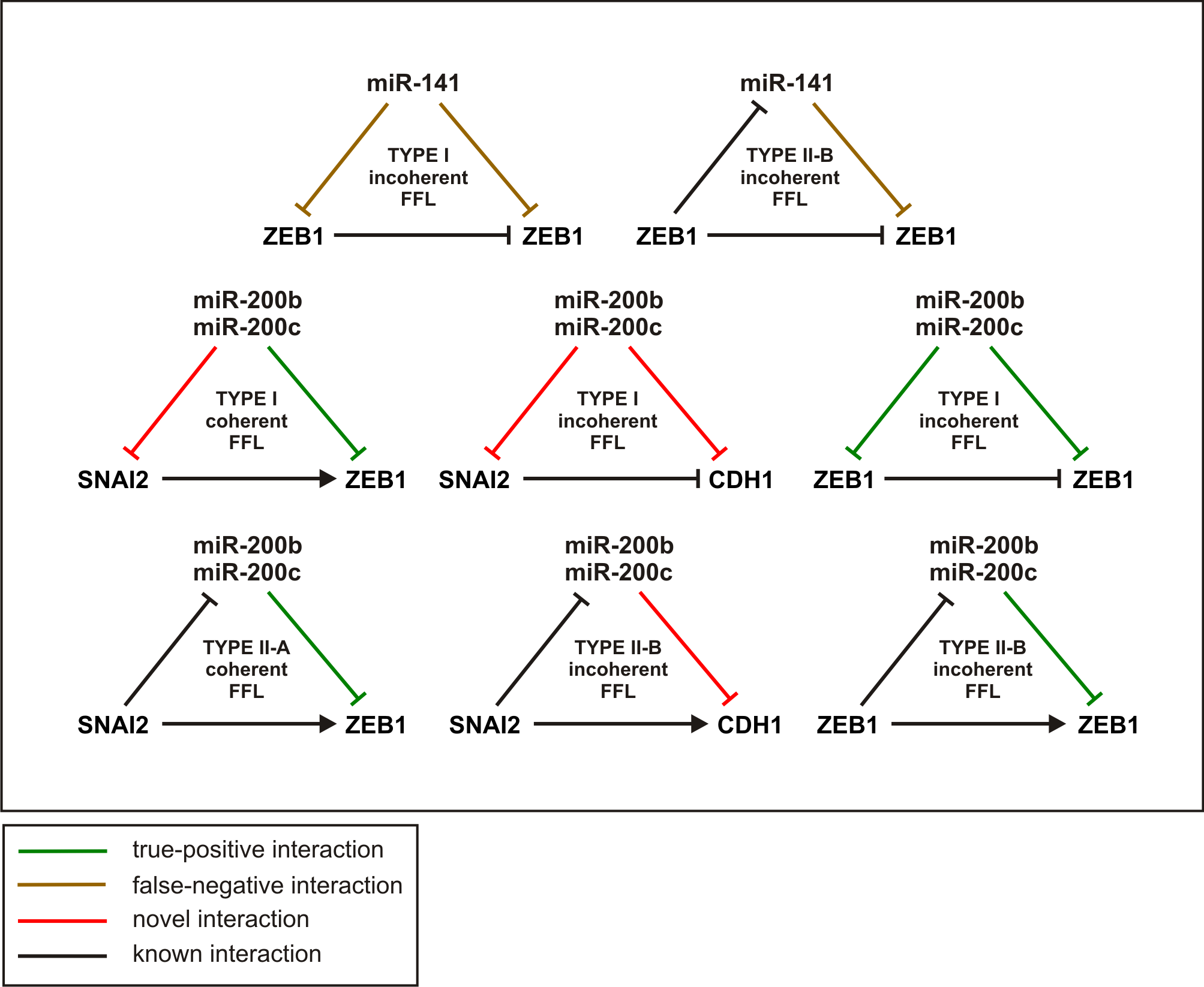

Supplement: Figure S2 — Deregulated FFLs predicted by IntegraMiR with nodes comprising only entries among miR-200b, miR-200c, and miR-141, as well as CDH1, SNAI2 (SLUG), and ZEB1. The FFLs are consistently deregulated based on the data. Green edges depict true-positive miRNA-target interactions identified by the predictive module of IntegraMiR, brown edges represent false-negative miRNA-target interactions, red edges depict novel miRNA-target interactions, and black edges depict known miRNA-target interactions. (TIF) [file pone.0100806.s002.tif]
