# Supplementary material for: Integrative Identification of Deregulated MiRNA/TF-Mediated Gene Regulatory Loops and Networks in Prostate Cancer
Source: PLoS One. 2014 Jun 26;9(6):e100806. doi: 10.1371/journal.pone.0100806 (PMC4072696; doi:10.1371/journal.pone.0100806)
Supplement: Table S13 — Lists of mRNAs, TFs, miRNAs, and their targets used to construct deregulated loops and rank their statistical significance. (PDF) [file pone.0100806.s015.pdf]

Supplementary Table S13 Lists of mRNAs, TFs, miRNAs, and their targets used to construct deregulated loops and rank their statistical significance.

<table>
<tr><td rowspan="2">MHT</td><td colspan="3">L_{mRNA}</td><td colspan="3">L_{miRNA}</td></tr>
<tr><td colspan="3">• mRNAs
• MHT FDR values
• direction of deregulation</td><td colspan="3">• miRNAs
• MHT FDR values
• direction of deregulation</td></tr>
<tr><td rowspan="2">GSEA</td><td colspan="2">$L_{\mathrm{TF}}^{\mathrm{GS}}$</td><td colspan="2">$L_{\mathrm{miRNA}}^{\mathrm{GS}}$</td><td colspan="2">$L_{\mathrm{KEGG}}^{\mathrm{GS}}$</td></tr>
<tr><td colspan="2">• TFs from mSigDB in L_{mRNA}
• MHT FDR values > 0.05
• GSEA FDR values ≤ 0.25
• direction of deregulation</td><td colspan="2">• miRNAs from miRTarBase in $L_{\mathrm{miRNA}}^{\mathrm{DeepSeq}}$
• MHT FDR values > 0.05
• GSEA FDR values ≤ 0.25
• direction of deregulation</td><td colspan="2">• KEGG pathways from mSigDB
• GSEA FDR values ≤ 0.25</td></tr>
<tr><td rowspan="4">TARGET IDENTIFICATION</td><td colspan="3">$L_{\mathrm{mRNA}}^{\mathrm{DiffExp}}$</td><td colspan="3">$L_{\mathrm{miRNA}}^{\mathrm{DiffExp}}$</td></tr>
<tr><td colspan="3">• mRNAs
• MHT FDR values ≤ 0.05
• MHT FDR values > 0.05 & GSEA FDR values ≤ 0.25
• direction of deregulation</td><td colspan="3">• miRNAs
• MHT FDR values ≤ 0.05
• MHT FDR values > 0.05 & GSEA FDR values ≤ 0.25
• direction of deregulation</td></tr>
<tr><td colspan="2">$L_{\mathrm{TF}}^{\mathrm{mRNA}}$</td><td colspan="2">$L_{\mathrm{TF}}^{\mathrm{miRNA}}$</td><td colspan="2">$L_{\mathrm{miRNA}}^{\mathrm{mRNA}}$</td></tr>
<tr><td colspan="2">• TFs in $L_{\mathrm{mRNA}}^{\mathrm{DiffExp}}$
• mRNA targets from TRANSFAC in L_{mRNA}
• regulation type</td><td colspan="2">• TFs in $L_{\mathrm{mRNA}}^{\mathrm{DiffExp}}$
• transcriptional miRNA targets from TransmiR in $L_{\mathrm{miRNA}}^{\mathrm{DiffExp}}$
• regulation type</td><td colspan="2">• miRNAs in $L_{\mathrm{miRNA}}^{\mathrm{DiffExp}}$
• predicted mRNA targets in L_{mRNA} using miRecords, amended with targets from miRTarBase</td></tr>
</table>
